# Supplementary figures and images for: The social media diet: A scoping review to investigate the association between social media, body image and eating disorders amongst young people
Source: PLOS Glob Public Health. 2023 Mar 22;3(3):e0001091. doi: 10.1371/journal.pgph.0001091 (PMC10032524; doi:10.1371/journal.pgph.0001091)

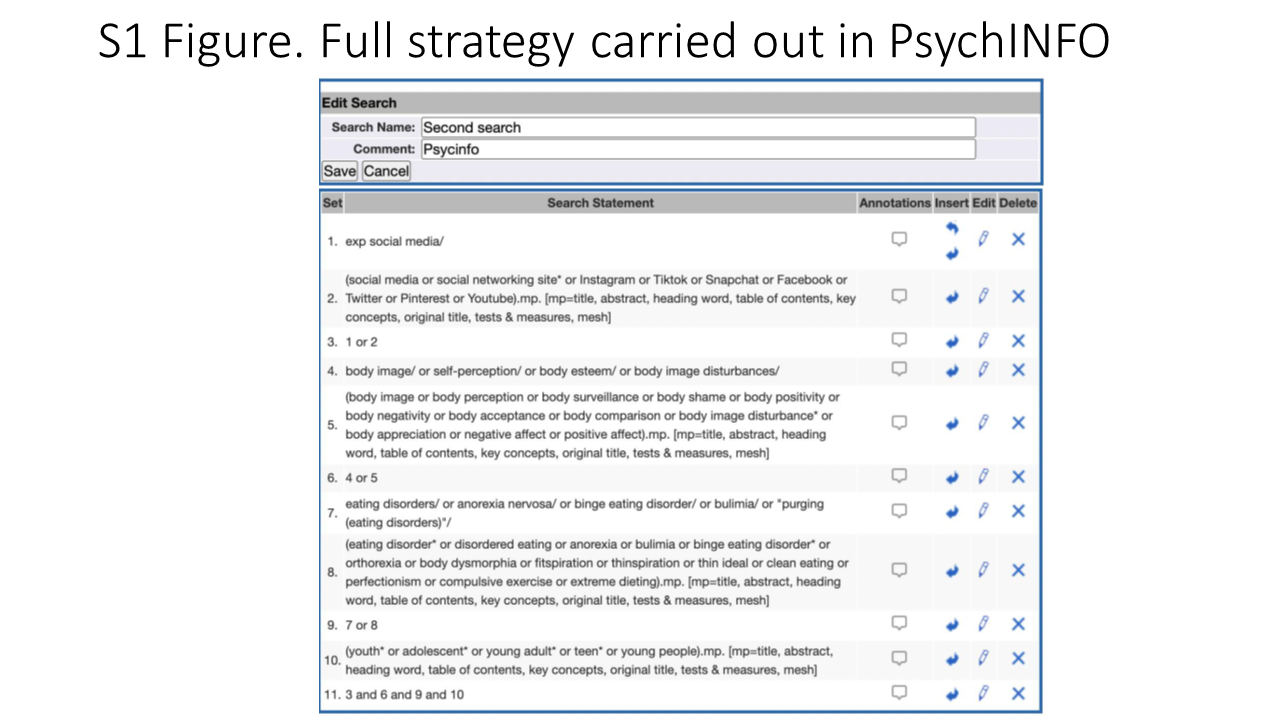

Supplement: S1 Fig — (TIF) [file pgph.0001091.s001.tif]
