# Supplementary material for: The social media diet: A scoping review to investigate the association between social media, body image and eating disorders amongst young people
Source: PLOS Glob Public Health. 2023 Mar 22;3(3):e0001091. doi: 10.1371/journal.pgph.0001091 (PMC10032524; doi:10.1371/journal.pgph.0001091)
Supplement: S1 Table — (PDF) [file pgph.0001091.s002.pdf]

S2 Table. Summaries of individual studies

#	Author	Country & World Bank Classification	Study Design	Participant characteristics		Objectives	Underlying theoretical framework	Social media platform	Exposure measure (Social media use)	Outcome measure (BI/ED symptomatology)	Mediator measure	Moderator measure	Results	Quality appraisal grade
**1.**	Brown and Tiggemann (2016)	Australia<br><br>High-income economy	Experimental	N (% F)<br>Setting:<br>Age range (mean +- SD)<br>BMI<br>Ethnicity<br>Sexual orientation<br>ED prevalence	138 (100% F)<br>University<br>18-30 (20.10 +- 2.61)<br>22.61<br>77.4% White<br>NR<br>NR	Investigated the impact of attractive celebrity and peer images on women's body image	Social comparison theory	IG	Randomly assigned to view attractive celebrity images, peer images or control set of travel images	Negative mood and BID: Heinberg and Thompson (1996) visual analogue scales	Appearance comparisons: The State Appearance Comparison Scale (Tiggemann and McGill 2004)	N/A	Exposure to celebrity & peer images both increased negative mood and BID relative to travel images with small/moderate effect size<br>Relationship mediated by appearance comparisons<br>Partly agrees with social comparison theory but participants compared despite being 'unknown' celebrities<br>Blurred line	Moderate

#	Author	Country & World Bank Classification	Study Design	Participant characteristics		Objectives	Underlying theoretical framework	Social media platform	Exposure measure (Social media use)	Outcome measure (BI/ED symptomatology)	Mediator measure	Moderator measure	Results	Quality appraisal grade
													between celebrities and peers- elevated bar due to editing features	
**2.**	Kim and Park (2016)	US<br><br>High-income economy	Experimental	N (% F)<br>Setting<br>Age range (mean +- SD)<br>BMI<br>Ethnicity<br><br><br><br><br><br>Sexual orientation<br>ED prevalence	116 (100% F)<br>University<br>NR (23+- 3.21)<br>23.79<br>38.8% White, 31.9% Asian, 10.3% African American, 5.2% Hispanic, 8.6% multiracial<br>NR<br>NR	Investigated the effects of FB News Feed photos on female student's appearance satisfaction	Social comparison theory	FB	FB News Feed exposure	BID: Visual analogue scales	N/A	Levels of appearance comparison orientation: Physical Appearance Comparison Scale<br><br>Self-esteem: Rosenberg self-esteem scale<br><br>Appearance schemas: Appearance schemas inventory (ASI)	Exposure to photographs of attractive females → less satisfaction with own BI relative to exposure to less attractive females on FB news feed<br>Mediated by social comparison (upward)<br>Moderated by appearance comparison orientation, appearance schemas (high) and self-esteem (low): heightens	Moderate

#	Author	Country & World Bank Classification	Study Design	Participant characteristics		Objectives	Underlying theoretical framework	Social media platform	Exposure measure (Social media use)	Outcome measure (BI/ED symptomatology)	Mediator measure	Moderator measure	Results	Quality appraisal grade
													vulnerability to effect	
3.	Murray et al (2016)	Canada	CS	N (% F)<br>Setting<br>Age range (mean +- SD)<br>BMI<br>Ethnicity<br>Sexual orientation<br>ED prevalence	388 (70.2% F)<br>University<br>NR (23.08 +- 3.09)<br>NR<br>52.2% White American, 12.9% European, 11.3% Asian, 8.4% Middle Eastern, 5.2% African, 37% Caribbean, 1.8% Central/South American, 42% Other<br>NR<br>NR	Investigated whether body image concerns (appearance & weight esteem) mediate the relationship between time spent on SNS and DE behaviours (restrained & emotional)	Social comparison theory<br><br>Sociocultural theory	General	Excessive SM use: adapted version of the generalised problematic internet use scale	DE: Disordered Eating Behaviours Questionnaire (DEBQ)	BI: The Body Esteem Scale for Adolescents and Adults	N/A	Excessive time on SM → diminished weight and appearance esteem → emotional eating for females only<br>Excessive time on SNS → diminished weight and appearance esteem → restrained eating for both genders<br>BI (appearance and weight esteem) mediated the relationship<br>Partly moderated by gender (emotional eating)	High

#	Author	Country & World Bank Classification	Study Design	Participant characteristics		Objectives	Underlying theoretical framework	Social media platform	Exposure measure (Social media use)	Outcome measure (BI/ED symptomatology)	Mediator measure	Moderator measure	Results	Quality appraisal grade
													Directionality of relationship not certain (i.e., SM → BI or BI → SM)	
**4.**	Puccio et al (2016)	Australia<br><br>High-income economy	Observational longitudinal	N (% F)<br>Setting<br>Age range<br>(mean +- SD)<br>BMI<br>Ethnicity<br>Sexual orientation<br>ED prevalence	245 (100% F)<br>University<br>NR (23.77 +- 7.10)<br>NR<br>NR<br>NR<br>NR	Investigated the mediating role of social comparisons and the moderating role of sociotrophy on the relationship between FB and BN symptoms	Dual pathway model	FB	FB usage	BN symptoms: Bulimia & food preoccupation scale from EAT-26<br><br>BID: Body parts satisfaction sale<br><br>Depressive symptoms: Centre for Epidemiologic Studies Depression scale<br><br>Dietary restraint: dieting subscale EAT-26	Social comparisons: 7 questions adapted from Fardouly and Vartanian (2015)<br><br>Thin ideal internalization: Ideal Body Stereotype Scale	Sociotrophy: Personal Style Inventory-11	BID is a risk factor for the development of BN, depression, and restrained eating- support for BI as a mediator<br>Personality trait sociotrophy strengthens this relationship – support for sociotrophy as a moderator<br>Supports the mediating role of social comparisons	High
**5.**	Tan et al	Singapore	CS	N (% F)	56 (94.5% F)	Investigated	Gratification's	Multiple (FB,	SM usage:	DE: EDE-Q 6.0	N/A	N/A	Those with	Moderat

#	Author	Country & World Bank Classification	Study Design	Participant characteristics		Objectives	Underlying theoretical framework	Social media platform	Exposure measure (Social media use)	Outcome measure (BI/ED symptomatology)	Mediator measure	Moderator measure	Results	Quality appraisal grade
	(2016)	 High-income economy		Setting<br>Age range (mean +- SD)<br>BMI<br>Ethnicity<br><br><br>Sexual orientation<br>ED prevalence	Clinical<br>NR (20.84 +- 6.67)<br>18.17<br>74.1% Chinese, 13.4% Malays, 9.2% Indians, 3.3% Other<br>NR<br>18.2% AN<br>23.6% binge-purging, 34.5% BN, 9.1%	relationship between SM use and severity of illness for ED patients presenting at Singapore General Hospital	theory	YT)	self-reported questionnaire	questionnaire to assess disordered eating in past 10 days			higher DE scores sought out content that perpetuated and maintained ED<br>YouTube as primary concern in seeking out food and exercise related materials (unregulated content)<br>Top 3 reasons for SM use: weight loss information, tips on hiding ED, finding support<br>Of those visiting FB ED groups, 50% stated that it helped to maintain their ED<br>70.9% reported comparing	e

#	Author	Country & World Bank Classification	Study Design	Participant characteristics		Objectives	Underlying theoretical framework	Social media platform	Exposure measure (Social media use)	Outcome measure (BI/ED symptomatology)	Mediator measure	Moderator measure	Results	Quality appraisal grade
													themselves to others on SM	
6.	Ahadzadeh et al (2017)	Malaysia<br><br>Upper-middle-income economy	CS	N (% F)<br>Setting<br>Age range (mean +- SD)<br>BMI<br>Ethnicity<br><br><br><br>Sexual orientation<br>ED prevalence	273 (62.3% F)<br>University<br>18-26 (20.09 +- NR)<br>21.03<br>Malay (7%), Chinese (83.9%), Indian (2.6%), Other (6.6%)<br>NR<br>NR	Investigated the effect of IG usage on BID and whether this is mediated by appearance self-schema and self-discrepancy<br>Investigated moderating role of self-esteem	Social comparison theory<br><br>Impression management theory	IG	IG photo exposure	BI: 9-item Body Areas Satisfaction Scale (BASS)	Self-schema: beliefs about appearance questionnaire<br><br>Self-discrepancy: The body image ideals questionnaire	Self-esteem: Rosenberg (1965) scale measuring self-worth	Exposure to IG photos led to BID<br>Mediated by self-schema (caring about own appearance) and self-discrepancy (gap between actual and ideal self)<br>Self-esteem moderated this – stronger relationship between IG use, self-schema & self-discrepancy for those with low self esteem	Moderate

#	Author	Country & World Bank Classification	Study Design	Participant characteristics		Objectives	Underlying theoretical framework	Social media platform	Exposure measure (Social media use)	Outcome measure (BI/ED symptomatology)	Mediator measure	Moderator measure	Results	Quality appraisal grade
**7.**	Burnette et al (2017)	US<br><br>High-income economy	Qualitative focus groups	N (% F)<br>Setting<br>Age range (mean +- SD)<br>BMI<br>Ethnicity<br><br><br>Sexual orientation<br>ED prevalence	38 (100% F)<br>Secondary school<br>12-14 (13.14 +- 0.72)<br>NR<br>86.1% White, 13.9% Black, 5.6% Hispanic, 2.8% Asian<br>NR<br>NR	Investigated the relationship between SM & BI in early adolescent girls using six focus groups	Social learning theory	General	SM usage: activities, duration, level of parental monitoring	Feelings towards BI	N/A	SM literacy<br>School environment	Generally, girls demonstrated high levels of SM literacy and resilience to negative impacts of SM use on BI<br>Endorsed some appearance concerns and comparisons with peers<br>Positive school environment and SM literacy education buffered against deleterious effects- girls learnt about artificiality of SM images, self-acceptance, body diversity	High
**8.**	Cohen et al (2017)	Australia<br><br>High-	CS	N (% F)<br>Setting<br>Age	259 (100% F)<br>University<br>18-29 (22.97	Investigated relationship between	Sociocultural theory	Multiple & IG	SM usage: The FB questionnaire	BI: Appearance evaluation subscale of the	Thin-ideal internalization:	N/A	Appearance focused SM use rather than	Moderate

#	Author	Country & World Bank Classification	Study Design	Participant characteristics		Objectives	Underlying theoretical framework	Social media platform	Exposure measure (Social media use)	Outcome measure (BI/ED symptomatology)	Mediator measure	Moderator measure	Results	Quality appraisal grade
		income economy		range (mean +- SD)<br>BMI<br><br>Ethnicity<br><br><br><br><br><br>Sexual orientation<br>ED prevalence	+- 3.25)<br>22.45<br>77.5% White, 15.1% Asian, 2.7% Middle Eastern, 0.8% African, 0.8% Aboriginal, 3.1% Other<br>NR<br>NR	appearance focused SM usage and BI concerns in young women	Objectification theory		to assess FB appearance exposure	Multidimensional Body-self relations questionnaire	Internalisation subscale of the sociocultural attitudes towards appearance questionnaire (SATAQ)<br><br>Appearance comparison: 5-item scale<br><br>Body surveillance: subscale of the Objectified body consciousness scale		overall use was related to BI concerns in young women<br>Thin ideal internalization and body surveillance mediated this relationship<br>Following appearance neutral accounts was not associated with BI outcomes<br>Effect size was stronger for IG compared to FB	
**9.**	Howard et al (2017)	US (Ahadzadeh, Pahlevan Sharif & Ong, 2017)	CS	N (% F)<br>Setting<br>Age (mean +- SD)	922 (100% F)<br>University<br>18-30 (21 +- 2.8)<br>25 White,	Investigated racial differences in SM use and BID and DE	Gratifications theory	Multiple: FB, IG, Twitter	SM usage: frequency	BID: The body shape questionnaire<br><br>DE: Eating	Investment: SM reassurance seeking scale	Race	Black women reported marginally less DE and higher BIS but small	Moderate

#	Author	Country & World Bank Classification	Study Design	Participant characteristics		Objectives	Underlying theoretical framework	Social media platform	Exposure measure (Social media use)	Outcome measure (BI/ED symptomatology)	Mediator measure	Moderator measure	Results	Quality appraisal grade
		High-income economy		BMI<br>Ethnicity<br>Sexual orientation<br>ED prevalence	28.24 Black<br>52% White, 48% Black<br>NR<br>NR					Disorder Examination Questionnaire (EDE-Q)			effect size<br>Engagement in reassurance seeking on FB (seeking likes and comments) mediated the relationship → more BID and DE for the entire sample<br>Strongest relationship found for FB	
10.	Kaewpradub et al (2017)	Thailand<br><br>Upper-middle-income economy	CS	N (% F)<br>Setting<br>Age range (mean +- SD)<br>BMI<br>Ethnicity<br>Sexual orientation<br>ED prevalen	620 (60.3% F)<br>Secondary school<br>NR (15.7 +- 1.9)<br>NR<br>NR<br>NR<br>NR but ½ had abnormal eating patterns	Investigated the association between time spent on SM, BI and eating behaviours	Sociocultural theory	General	SM usage: time spent, exposure to certain content	BI: Thai Body Esteem Scale for Adolescents and Adults<br><br>Drive for muscularity scale<br><br>ED attitudes and behaviour: Thai version of the EAT-26	N/A	Gender	Use of SM was negatively associated with greater BID and time spent using SM was associated with binging, purging, use of laxative/weight loss or diuretics and eating behaviours	Moderate

#	Author	Country & World Bank Classification	Study Design	Participant characteristics		Objectives	Underlying theoretical framework	Social media platform	Exposure measure (Social media use)	Outcome measure (BI/ED symptomatology)	Mediator measure	Moderator measure	Results	Quality appraisal grade
				ce									carrying risk of obesity e.g., crash diets.<br>½ of participants demonstrated abnormal eating behaviours<br>Gender was a moderator-female participants had greater BID compared to men	
11.	Rousseau et al (2017)	Belgium<br><br>High-income economy	Observational longitudinal	N (% F)<br>Setting<br>Age range (mean +-SD)<br>BMI<br>Ethnicity<br>Sexual orientation<br>ED	1840 (48% F)<br>Secondary school<br>12-19 (14.76+-1.41)<br>NR<br>NR<br>NR<br>NR	Investigated whether passive FB use is related to adolescents BID through comparison over 6 months	Social comparison theory<br><br>Tripartite influence model<br><br>Gratification's theory	FB	FB usage: time spent<br><br>Passive FB usage: Passive FB use subscale of the multidimensional scale of FB use	BID: Subscale of the Body Attitude Test	Social comparison: frequency assessed using 5-point Likert scale	Gender<br><br>Pre-existing BID	Significant relationship between passive FB use → appearance comparisons → BID for boys only<br>Comparison on Facebook was not predictive of increases in BID at 6 months	Moderate

#	Author	Country & World Bank Classification	Study Design	Participant characteristics		Objectives	Underlying theoretical framework	Social media platform	Exposure measure (Social media use)	Outcome measure (BI/ED symptomatology)	Mediator measure	Moderator measure	Results	Quality appraisal grade
				prevalence									For both genders, state BID predicted comparisons on FB- suggestive that body dissatisfied individuals seek out comparison content when using FB	
12.	Santarossa and Woodruff (2017)	Canada<br><br>High-income economy	CS	N (% F)<br>Setting<br>Age range (mean +- SD)<br>BMI<br>Ethnicity<br>Sexual orientation<br>ED prevalence	212 (55% F)<br>University<br>18-27 (85% 18-19)<br>NR<br>NR<br>NR<br>NR	Investigated whether problematic SM use was related to BI, self esteem and ED symptoms/concerns	Sociocultural theory	MultipleTwitter, IG, Pinterest	SM usage: time spent, total friends, specific SM activities<br><br>Problematic SM use: 29 item generalised problematic internet use scale	ED: EAT-26 scale<br><br>Self-esteem: The Rosenberg Self-Esteem Scale	N/A	N/A	Problematic SM use was significantly associated with higher BID, lower self-esteem and higher ED symptoms/concerns<br>SM investment i.e., looking at own profile, looking at others, leaving comments was	Moderate

#	Author	Country & World Bank Classification	Study Design	Participant characteristics		Objectives	Underlying theoretical framework	Social media platform	Exposure measure (Social media use)	Outcome measure (BI/ED symptomatology)	Mediator measure	Moderator measure	Results	Quality appraisal grade
													strongly related to BI<br>Overall SM use related to ED symptomatology<br>Gender was not a moderator	
13.	Turner and Lefevre (2017)	UK<br><br>High-income economy	CS	N (% F)<br>Setting<br>Age range (mean +- SD)<br>BMI<br>Ethnicity<br>Sexual orientation<br>ED prevalence	713 (96% F)<br>Online<br>18-75 (24.96 +- 8.17)<br>22.14 (H)<br>NR<br>NR<br>49% met diagnostic criteria for ON	Investigated the association between IG and orthorexia nervosa symptoms amongst a population that followed health accounts	Cultivation theory<br><br>Gratification's theory	IG	SM usage: frequency & duration	Dietary choices: which of 19 foods were included in their diet<br><br>ON: ORTO-16 questionnaire	N/A	N/A	Higher IG was significantly associated with ON (49% prevalence amongst participants compared to <1% amongst general population)<br>Small effect size but significant at population level<br>80% IG users ranked food as first or second most frequent image category	High

#	Author	Country & World Bank Classification	Study Design	Participant characteristics		Objectives	Underlying theoretical framework	Social media platform	Exposure measure (Social media use)	Outcome measure (BI/ED symptomatology)	Mediator measure	Moderator measure	Results	Quality appraisal grade
													appearing on IG feed<br>Reference to IG 'health celebrities' with large followings, damaging dietary advice, obsessive exercise regimes<br>Age did not moderate the relationship	
14.	Easton et al (2018)	UK<br><br>High-income economy	Qualitative focus groups	N (% F)<br>Setting<br>Age range (mean +- SD)<br>BMI<br>Ethnicity<br>Sexual orientation<br>ED prevalen	20 (70% F)<br>University<br>18-25 (20.7, NR)<br>NR<br>NR<br>NR<br>NR	Investigated experiences of Fitspiration amongst users: impact on BI, emotions, behaviour and health	Social comparison theory<br><br>Impression management theory	MultipleIG, FB, YT	SM Fitspiration exposure	BI (thoughts, emotions, behaviour, health)	N/A	N/A	4 themes arose from qualitative analysis: 1) supporting positive behaviour change 2) Unrealistic and untrustworthy content 3) negative effects on emotional wellbeing 4)	High

#	Author	Country & World Bank Classification	Study Design	Participant characteristics		Objectives	Underlying theoretical framework	Social media platform	Exposure measure (Social media use)	Outcome measure (BI/ED symptomatology)	Mediator measure	Moderator measure	Results	Quality appraisal grade
				ce									vulnerabilities Revealed that Fitspiration can be motivating but elicits comparisons, exposure to unattainable and dangerous content, and has serious consequences for mental health Effects may persist despite critical awareness of 'damaging' posts This relationship was stronger for girls	
15.	Kleemans et al 2018)	The Netherlands<br><br>High-	Mixed Methods: online experiment	N (% F)<br>Setting<br>Age range	144 (100% F)<br>Secondary school<br>NR (15.92 +-	Investigated the effect of manipulated IG photos on	Social comparison theory	IG	Exposure to manipulated photos on IG	BI: The Body Image State scale	N/A	Social comparison tendencies: The	Exposure to manipulated IG photos → BID Stronger	Moderate

#	Author	Country & World Bank Classification	Study Design	Participant characteristics		Objectives	Underlying theoretical framework	Social media platform	Exposure measure (Social media use)	Outcome measure (BI/ED symptomatology)	Mediator measure	Moderator measure	Results	Quality appraisal grade
		income economy	& survey	(mean +- SD)<br>BMI<br>Ethnicity<br>Sexual orientation<br>ED prevalence	1.16)<br>NR<br>NR<br>NR<br>NR	adolescent girls BI and whether social comparison tendency moderates the relationship	Impression management theory					Netherlands Comparison Orientation Measure	relationship for girls with higher social comparison tendencies (moderating factor)<br>Girls demonstrated little awareness of manipulated photos and rated them as more attractive in comparison to original photos – editing as accepted and normalised for contemporary teenagers	
16.	Marengo et al (2018)	Italy<br><br>High-income economy	CS	N (% F)<br>Setting<br>Age (mean +- SD)<br>BMI	523 (53.5% F)<br>Secondary school<br>NR (14.82 +- 1.52)<br>NR	Investigated the association SM use (appearance focused), BI concerns and internalizing	Sociocultural theory<br><br>Social comparison theory	MultipleIG, SC & FB	Appearance focused SM exposure	BI: Italian version of the Body Shape Questionnaire<br><br>Internalizing	N/A	N/A	Students report use of appearance focused SM for >2 hours per day → significantly	Moderate

#	Author	Country & World Bank Classification	Study Design	Participant characteristics		Objectives	Underlying theoretical framework	Social media platform	Exposure measure (Social media use)	Outcome measure (BI/ED symptomatology)	Mediator measure	Moderator measure	Results	Quality appraisal grade
				Ethnicity<br>Sexual orientation<br>ED prevalence	NR<br>NR<br>NR	symptoms (anxiety and depression) amongst adolescents				symptoms: Italian self-rated version of the strength and difficulties questionnaire			higher BI concerns and internalising symptoms<br>This relationship was stronger for IG and SC in contrast to FB<br>Supports internalizing symptoms as an outcome comorbid with BI concerns although directionality cannot be assumed	
17.	Raggatt et al (2018)	Australia<br><br>High-income economy	Mixed Methods: CS survey and open-ended questionnaire	N (% F)<br>Setting<br>Age (mean +- SD)<br>BMI<br>Ethnicity<br>Sexual orientatio	180 (84% F)<br>Online<br>NR (23 +- NR)<br>24.5 (H)<br>NR<br>NR<br>17.7% high risk of ED	Investigated the association between fitspiration, DE, exercise behaviours and psychological distress of fitspiration	Sociocultural<br><br>Social identity<br><br>Gratification's theory	General	SM Fitspiration exposure: checklist indicating type of fitspiration content and engagement levels e.g.,	DE: EAT-26<br><br>Compulsive exercise behaviours: Exercise Addiction Inventory	N/A	N/A	17.7% classified as high risk of an ED, 17.4% reported high levels of psychological distress, 10.3% at risk of addictive	Moderate

#	Author	Country & World Bank Classification	Study Design	Participant characteristics		Objectives	Underlying theoretical framework	Social media platform	Exposure measure (Social media use)	Outcome measure (BI/ED symptomatology)	Mediator measure	Moderator measure	Results	Quality appraisal grade
				n<br>ED prevalence		followers/users			comment, posting, scrolling	Psychological distress: Kessler 10 Psychological Distress Scale			exercise behaviours<br>Positive engagement: motivation, goal setting, strength, community, reliable health information, food & exercise inspiration<br>Negative: failing to meet the ideal, anxiety, hampering ED recovery, pressure of clean eating and exercise, underqualified advice	
18.	Saunders and Eaton (2018)	USA<br><br>High-income economy	CS	N (% F)<br>Setting<br>Age range (mean +-SD)	637 (100% F)<br>University<br>18-24 (21.3 +- 1.72)<br>NR<br>67.5%	Investigated the sociocultural model of eating with theories explaining the impact of SM on	Sociocultural<br><br>Social comparison theory	Multiple: IG, SC & FB	SM use: self-report survey on motivation for usage, time spent	DE: Eating Pathology Symptoms Inventory	Appearance comparisons: Upward and downward comparison	N/A	Significant positive relationship between upward comparison and DE outcomes	Moderate

#	Author	Country & World Bank Classification	Study Design	Participant characteristics		Objectives	Underlying theoretical framework	Social media platform	Exposure measure (Social media use)	Outcome measure (BI/ED symptomatology)	Mediator measure	Moderator measure	Results	Quality appraisal grade
				BMI<br>Ethnicity<br><br><br><br><br><br>Sexual orientation<br>ED prevalence	Hispanic white, 12.4% non-Hispanic white, 12.6% Black, 2.5% Asian, 3.5% multiracial, 1.6% Other<br>NR<br>NR	the development of DE for users of three SM platforms	Objectification theory<br><br>Cultivation theory				scale<br><br>Body surveillance: body surveillance subscale of the Objectified Body Consciousness Scale		and between body surveillance and DE outcomes (mediators)<br>Stronger relationships for IG & SC (appearance focused platforms)<br>Significant association between BID, binge eating and cognitive restraint (supports BI as a mediator)<br>IG as the most popular platform to be seen by others	
19.	Tiggemann et al (2018)	Australia<br><br>High-income	Experimental	N (% F)<br>Setting<br>Age range	220 (100% F)<br>University<br>NR (20.13 +- 2.58)	Investigated the relationship between SM investment	Social comparison theory	IG	SM use: self-report survey<br><br>Exposure to	Body & facial dissatisfaction: Heinberg and Thompson	Appearance comparison: Tiggemann and McGill	N/A	Number of likes had no impact on appearance comparison,	Moderate

#	Author	Country & World Bank Classification	Study Design	Participant characteristics		Objectives	Underlying theoretical framework	Social media platform	Exposure measure (Social media use)	Outcome measure (BI/ED symptomatology)	Mediator measure	Moderator measure	Results	Quality appraisal grade
		economy		(mean +-SD)<br>BMI<br>Ethnicity<br><br><br><br>Sexual orientation<br>ED prevalence	23.40 (H)<br>67.5% White, 23.2% Asian, 2.3% African, 1.4% Aboriginal, 5.9% 'other'<br>NR<br>NR	('likes' on IG) and women's BI	Impression management theory<br><br>Gratification's theory		IG photos	(1995) visual analogue scale	(2004) State Appearance Comparison Scale		body dissatisfaction or facial dissatisfaction but led to facial satisfaction BUT investment in likes → appearance comparison → facial dissatisfaction (Supports gratification theory)<br>There was no impact on BID	
20.	Aparicio-Martinez et al (2019)	Spain<br><br>High-income economy	CS	N (% F)<br>Setting<br>Age range (mean +-SD)<br>BMI<br>Ethnicity<br>Sexual orientatio	168 (100% F)<br>University<br>18-25 (20 +-0.76)<br>NR<br>96.7% White<br>NR<br>NR but 28.5% demonstrated DE attitudes	Investigated the relationship between SM usage, BI and whether this is moderated by levels of testosterone	Social comparison theory<br><br>Objectification theory	Multiple<br>Twitter, FB, IG, YT, SC	SM use: self-report survey	DE: EAT-26 tool<br><br>BI: Body satisfaction questionnaire<br><br>SE: Rosenberg survey for self esteem	N/A	Prenatal testosterone	28.5% of females had DE attitudes: 38.7% presented BN symptoms and 20.5% reported dieting<br>DE attitudes were significantly associated with	Moderate

#	Author	Country & World Bank Classification	Study Design	Participant characteristics		Objectives	Underlying theoretical framework	Social media platform	Exposure measure (Social media use)	Outcome measure (BI/ED symptomatology)	Mediator measure	Moderator measure	Results	Quality appraisal grade
				n<br>ED prevalence									SE, BI, desired body (93% wanted to change 3 zones of body) and SM use- directionality not assumed<br>Addiction to SM (problematic use) &<br>Prenatal testosterone was a moderator: higher levels → decreased association between SM use, BID and DE behaviours<br>Self-esteem and thin ideal internalization as plausible mediators	
21.	Baker et al (2019)	US	Qualitative focus	N (% F)<br>Setting	27 (100% F)<br>University	Investigated the association	Sociocultural theory	IG	IG usage: self-reported	Perceptions related to IG	N/A	N/A	Students heavily invested in SM	Moderate

#	Author	Country & World Bank Classification	Study Design	Participant characteristics		Objectives	Underlying theoretical framework	Social media platform	Exposure measure (Social media use)	Outcome measure (BI/ED symptomatology)	Mediator measure	Moderator measure	Results	Quality appraisal grade
		High-income economy	groups	Age (mean +- SD)<br>BMI<br>Ethnicity<br>Sexual orientation<br>ED prevalence	18-22 (20 +- 1.2)<br>NR<br>63% White American<br>NR<br>NR	between IG use and BI amongst university students	Gratification's theory<br><br>Objectification theory		and discussed	usage, importance of specific functions e.g., likes & body image			posts, selected the most flattering photos, edited others, sought out 'likes', and deleted photos when they did not receive enough Demonstrated pressure to adhere to societal beauty ideals, comparison with other IG users, imitating photos of 'attractive' others, feeling ugly, placing greater emphasis on online identity, internalising 3rd person perspective of	

#	Author	Country & World Bank Classification	Study Design	Participant characteristics		Objectives	Underlying theoretical framework	Social media platform	Exposure measure (Social media use)	Outcome measure (BI/ED symptomatology)	Mediator measure	Moderator measure	Results	Quality appraisal grade
													the self	
22.	Chang et al (2019)	Singapore<br><br>High-income economy	CS	N (% F)<br>Setting<br>Age (mean +- SD)<br>BMI<br>Ethnicity<br><br>Sexual orientation<br>ED prevalence	303 (100% F)<br>Secondary school<br>12-16 (NR)<br>NR<br>Chinese (72%), Malay (14%), Indian (8%), Eurasian (1%), Other (1%)<br>NR<br>NR	Investigated the association between adolescents' girls IG selfie* practices and BI<br>Mediating role of appearance comparisons was examined	Social comparison theory<br><br>Objectification theory<br><br>Impression management theory<br><br>Social identity theory	IG	Selfie practices on IG: three types of selfie related activities explored	BI: Mendelson, White & Mendelson's Body Esteem Scale	Peer appearance comparisons: three items adapted from the Thompson, Heinberg and Tantleff-Dunn's Physical Comparison Scale	Direction of appearance comparisons: two items adapted from Fardouly and Vartanian (2015)	Photo browsing → negative body esteem which was mediated by peer comparison<br>Photo posting → positive body esteem (posting 'best image' of oneself and receiving positive peer feedback may boost confidence and BI)<br>Peer comparisons mediated both relationships<br>Direction of comparison was not a moderator	Moderate
23.	De Vries et al (2019)	The Netherlands	CS	N (% F)<br>Setting<br>Age	440 (47% F)<br>Secondary school	Investigated the association between SM use	Sociocultural theory	FB	SM use: Adapted version of the	BID: Body dissatisfaction subscale of the	N/A	Positive mother/father-adolescent	SM use was significantly associated with	High

#	Author	Country & World Bank Classification	Study Design	Participant characteristics		Objectives	Underlying theoretical framework	Social media platform	Exposure measure (Social media use)	Outcome measure (BI/ED symptomatology)	Mediator measure	Moderator measure	Results	Quality appraisal grade
		High-income economy		(mean +- SD)<br>BMI<br>Ethnicity<br>Sexual orientation<br>ED prevalence	12-19 (14.86 +- 1.79)<br>NR<br>NR<br>NR<br>NR	and BI and whether gender and positive mother/father-adolescent relationships moderated the relationship			Multidimensional Scale of FB use	Body Attitude Test		relationships: The Network of Relationships Questionnaire<br><br>Gender	BID, but this relationship was weaker for adolescents with positive mother-adolescent relationships (moderator)<br>Positive father-adolescent relationships did not moderate the association<br>Gender was not a significant moderator	
24.	Lonergan et al (2019)	Australia<br><br>High-income economy	CS	N (% F)<br>Setting<br>Age (mean +- SD)<br>BMI<br>Ethnicity<br>Sexual orientation<br>ED	184 (52.6% F)<br>University<br>17-40 (20.13+-3.43)<br>20.13 (H)<br>NR<br>NR<br>NR	Investigated the relationship between manipulation of selfies, investment in other's responses to selfies & BID<br>Also explored if self-compassion	Impression management theory<br><br>Gratification's theory	General	SM use: self-report usage and activities	BID: 16-item self-report adapted version of the Body Shape Satisfaction Scale	Photo manipulation and photo investment: Photo manipulation and investment scales	Self-compassion: The 12-item Self-Compassion Scale Short-Form	SM photo manipulation and investment was significantly associated with BID for both genders, but directionality of relationship not assumed (i.e. BID → SM	High

#	Author	Country & World Bank Classification	Study Design	Participant characteristics		Objectives	Underlying theoretical framework	Social media platform	Exposure measure (Social media use)	Outcome measure (BI/ED symptomatology)	Mediator measure	Moderator measure	Results	Quality appraisal grade
				prevalence		moderated the relationship							photo manipulation and investment or SM photo manipulation and investment → BID)<br>Self-compassion did not moderate the relationship	
25.	Wiklund et al (2019)	Sweden<br><br>High-income economy	Qualitative: interviews	N (% F)<br>Setting<br>Age (mean +- SD)<br>BMI<br>Ethnicity<br>Sexual orientation<br>ED prevalence	36 (NR)<br>Secondary school<br>16-18 (17.7 +- NR)<br>NR<br>NR<br>NR<br>NR	Explored the topic of SM in relation to norms, ideals regarding body appearance, exercise, and knowledge of ON	Sociocultural theory	General	SM use: self-report during interviews	Perceptions of healthism, social media, norms, ideals regarding body image and knowledge of ON	N/A	N/A	SM as setting the standard: promoting unattainable fitness standards, thin/fit ideal, fitness fixation, extreme exercise, and competition<br>Adhering as part of a quest for social recognition, value, and	High

#	Author	Country & World Bank Classification	Study Design	Participant characteristics		Objectives	Underlying theoretical framework	Social media platform	Exposure measure (Social media use)	Outcome measure (BI/ED symptomatology)	Mediator measure	Moderator measure	Results	Quality appraisal grade
													status- achieved through the body<br>Falling short of the ideal → alienation, imperfection<br>Pressure for girls to appear slim and toned BUT not too muscular (aesthetic goals of 'fitspiration')	
26.	Bennettt et al (2020)	US<br><br>High-income economy	EMA	N (% F)<br>Setting<br>Age (mean +- SD)<br>BMI<br>Ethnicity<br><br><br>Sexual orientation<br>ED prevalence	30 (100% F)<br>University<br>NR (18.52 +- 0.87)<br>22.37 (H)<br>89.7% White, Hispanic (3.4%), Asian American/Asian (3.4%)<br>NR<br>NR	Investigated the effects of SM consumption on BID and negative affect using EMA	Sociocultural theory<br><br>Social comparison theory	General	SM consumption: EMA-compatible social media consumption questionnaire (time spent and social media type consumed)	BI: The Body Image States Scale	N/A	N/A	Number of SM sites visited was a significant predictor of BID whilst time spent using SM was not<br>Both number of sites visited, and time spent on SM were significant predictors of negative affect, sadness, and	Moderate

#	Author	Country & World Bank Classification	Study Design	Participant characteristics		Objectives	Underlying theoretical framework	Social media platform	Exposure measure (Social media use)	Outcome measure (BI/ED symptomatology)	Mediator measure	Moderator measure	Results	Quality appraisal grade
													guilt<br>100% of participants endorsed SM use	
27.	Cavazos-Rehg et al (2020)	US<br><br>High-income economy	Mixed methods	N (% F)<br>Setting<br>Age (mean +- SD)<br>BMI<br>Ethnicity<br><br>Sexual orientation<br>ED prevalence	598 (77.6% F)<br>Online<br>NR (19.77 +- 5.03)<br>NR<br>69.06% White American<br>NR<br>59.8% subclinical, 23.7% clinical	Investigated the self-reported advantages and disadvantages of SM related to BI/EDs and the openness of participants to online outreach and ED support. All participants endorsed posting or following the fit/thin ideal on SM	Sociocultural theory	MultipleTwitter, FB, SC, IG, Tumblr, YT, Pintere-st	Exposure to thin ideal content on SM: self-report questionnaire on posting, following, engagement in past month 96% had seen content, 96% followed it and 71% posted about it	DE: Stanford Fashington ED screening based on DSM-5 criteria<br><br>Social support questionnaire	N/A	N/A	General advantages: motivation to be thin, engage in exercise, work out, restrict eating, recover, and seek treatment (2.9%) Amongst the clinical or subclinical ED group – higher proportion of people claimed that the motivation to be thin and restrict diet compared to groups without an ED (vulnerability). All	Moderate

#	Author	Country & World Bank Classification	Study Design	Participant characteristics		Objectives	Underlying theoretical framework	Social media platform	Exposure measure (Social media use)	Outcome measure (BI/ED symptomatology)	Mediator measure	Moderator measure	Results	Quality appraisal grade
													participants reported that SM is risk for ED symptomatology and pressure for thinness.<br><br>General disadvantages: content elicits low self-esteem (61.31%), mentioning anxiety or depression, dealing with other reactions e.g., fear of judgement, lack of comments and likes, trigger to engage in ED behaviour, pressure to be thin, engaging in body comparisons,	

#	Author	Country & World Bank Classification	Study Design	Participant characteristics		Objectives	Underlying theoretical framework	Social media platform	Exposure measure (Social media use)	Outcome measure (BI/ED symptomatology)	Mediator measure	Moderator measure	Results	Quality appraisal grade
													obsessing over body size.	
28.	Choukas-Bradley et al (2020)	US<br><br>High-income economy	CS	N (% F)<br>Setting<br>Age (mean +-SD)<br>BMI<br>Ethnicity<br><br><br><br>Sexual orientation<br>ED prevalence	226 (58.4%)<br>Secondary school<br>NR (16.25 +-NR)<br>NR<br>45.6% White American, 24.3% African American, 25.2% Hispanic<br>NR<br>NR	Investigated the association between Appearance Related Social Media Consciousness* and DE and depressive symptoms	Sociocultural theory<br><br>Objectification theory	General	SM usage: self-reported time spent	DE: EDE-QS questionnaire<br><br>Depression: Depressive symptoms questionnaire	Body comparison: BEECOM scale<br><br>Appearance related social media consciousness scale<br><br>Body surveillance and body shame: Objectified Body Consciousness Scale<br><br>Self-Objectification: Self	Gender	Higher ARSMC → higher depressive symptoms for girls and boys<br>Higher ARSMC → DE amongst girls only<br>96% girls and 91% boys reported engaging in ARSMC on SM- curating photos, scrutinizing them, imaging how one appears to others- supporting body surveillance and self-objectification as	Moderate

#	Author	Country & World Bank Classification	Study Design	Participant characteristics		Objectives	Underlying theoretical framework	Social media platform	Exposure measure (Social media use)	Outcome measure (BI/ED symptomatology)	Mediator measure	Moderator measure	Results	Quality appraisal grade
											Objectification Behaviours and Belief scale		a plausible mediator	
29.	Fitzsimmons-Craft et al (2020)	US<br><br>High-income economy	CS	N (% F)<br>Setting<br>Age range (mean +- SD)<br>BMI<br>Ethnicity<br><br>Sexual orientation<br>ED prevalence	408 (100% F)<br>Online<br>15-25 (59% 15-17 years +- NR)<br>NR<br>65% White American<br>NR<br>84% met criteria for clinical/subclinical EDs and all engaged with pro-ED content on SM	Investigated exposure to BI content emphasising the thin ideal on SM and probably ED diagnosis ED-related QOL and psychiatric comorbidities (depression & anxiety)	Sociocultural theory	MultipleTwitter, FB, SC, IG, Tumblr, YT, Reddit	Thin ideal exposure on SM: three-item self-report scale	DE: Stanford Washington ED Screening and the ED Quality Of Life Instrument<br><br>Psychological comorbidities: Patient Health Questionnaire	N/A	Age	96% followed at least one account that posted thinspiration content, 96% saw peers post about eating/weight/BI, 72% posted about the thin-ideal<br>84% met the criteria for clinical/subclinical ED but only 14% had received treatment for eating problems in the past	Moderate

#	Author	Country & World Bank Classification	Study Design	Participant characteristics		Objectives	Underlying theoretical framework	Social media platform	Exposure measure (Social media use)	Outcome measure (BI/ED symptomatology)	Mediator measure	Moderator measure	Results	Quality appraisal grade
													month (fear of judgement, lack of trust, problem not perceived as serious enough)<br>71% reported symptoms consistent with depression & 65% of moderate anxiety<br>Strong support for the relationship between pro-ED content and ED diagnoses	
30	Gioia et al (2020)	Italy<br><br>High-income economy	CS	N (% F)<br>Setting<br>Age range (mean +- SD)<br>BMI<br>Ethnicity<br>Sexual orientatio	693 (55% F)<br>Secondary school<br>13-19 (19 +- NR)<br>NR<br>NR<br>NR<br>NR	Investigated the association between problematic social media use, body image control in photos (manipulation) and body shame (tested as an	Objectification theory<br><br>Impression management theory	General	Problematic SM usage: self-reported	Body Shame: 8-item Body Shame subscale of the Italian Objectified Body Consciousness Scale	Body image control: Body Image Control in Photos-Revised Scale (BICP-R)	Gender<br><br>Pre-existing Body Shame: 8-item Body Shame subscale of the Italian Objectified	Body shame significantly predicted image control in SM photos and engagement in Problematic SM use for both males and females	Low

#	Author	Country & World Bank Classification	Study Design	Participant characteristics		Objectives	Underlying theoretical framework	Social media platform	Exposure measure (Social media use)	Outcome measure (BI/ED symptomatology)	Mediator measure	Moderator measure	Results	Quality appraisal grade
				n ED prevalence		outcome and a moderator)						Body Consciousness Scale	Hypothetically implies that body shame → Problematic SM use and photo investment - - - > Self objectification BUT directionality cannot be assumed (supports potential bidirectional relationship) Young women had higher rates of body shame, engaged in more problematic SM use, and invested heavily in self-portraits	
31.	Krug et al	Australia	EMA	N (% F)	85 (100% F)	Investigated the	Tripartite	IG	Exposure to	Mood: single	Thin ideal	Trait body	Exposure to	High

#	Author	Country & World Bank Classification	Study Design	Participant characteristics		Objectives	Underlying theoretical framework	Social media platform	Exposure measure (Social media use)	Outcome measure (BI/ED symptomatology)	Mediator measure	Moderator measure	Results	Quality appraisal grade
	(2020)	High-income economy		Setting<br>Age range (mean +- SD)<br>BMI<br>Ethnicity<br><br>Sexual orientation<br>ED prevalence	Community<br>NR (19.68 +- 3.06)<br>22.06 (H)<br>41.2% White, 44.7% Asian, 4.7% Mediterranean, 2.4% African<br>NR<br>NR	impact of fitspiration images (relative to neutral) on BI, mood and DE and whether trait BID, thin-ideal internalization and pressures from media, family & peers moderated these effects	influence model		fitspiration images using EMA	item approach with responses on 10-point scale<br><br>DE: engagement in DE behaviours adapted from previous EMA studies	internalization: The Sociocultural Attitudes towards appearance questionnaire (SATAQ-4)<br><br>Appearance comparison: The Physical Appearance and Comparison Scale-Revised (PACS-R)	dissatisfaction: Body Image Dissatisfaction subscale of the body change inventory (BCI)<br><br>Pressures from media, family, and peers: self-reported<br><br>Thin/fit ideal internalization: self-reported	fitspiration images led to significantly higher perceived pressure to attain an idealised physique and decreased satisfaction with current fitness<br>Led to no significant effect on mood or disordered eating	
32.	Lonergan et al (2020)	Australia<br><br>High-income economy	CS	N (% F)<br>Setting<br>Age (mean +- SD)<br>BMI	4,209 (53.13% F)<br>Secondary school<br>11-19 (14.97 +- NR)	Investigated whether SM behaviours were associated with higher odds of meeting criteria	Impression management theory	Multiple (image-based)	SM usage: self-report on image-based SM usage	ED diagnosis: Eating Disorder Examination Questionnaire (EDE-Q version 6)	Social media activities: avoidance of posting selfies, photo	Gender	Found that each SM behaviour was associated with greater likelihood of meeting criteria	High

#	Author	Country & World Bank Classification	Study Design	Participant characteristics		Objectives	Underlying theoretical framework	Social media platform	Exposure measure (Social media use)	Outcome measure (BI/ED symptomatology)	Mediator measure	Moderator measure	Results	Quality appraisal grade
				Ethnicity<br>Sexual orientation<br>ED prevalence	64.4% boys & 67.3% girls in 'healthy' percentile<br>NR<br>NR<br>NR	for an ED & whether gender moderated this relationship				Psychological distress: K10 Psychological Distress Scale	investment, photo manipulation, investment in other's selfies		for one or more EDs (clinical and subclinical BN and night eating syndrome) when adjusting for demographic factors<br>Suggests that effort into choosing a selfie, editing images and monitoring comments & likes are pervasive SM behaviours in adolescents who meet the criteria for an ED – ED symptomatology (overvaluation) manifesting virtually in SM behaviours<br>Suggestive that	

#	Author	Country & World Bank Classification	Study Design	Participant characteristics		Objectives	Underlying theoretical framework	Social media platform	Exposure measure (Social media use)	Outcome measure (BI/ED symptomatology)	Mediator measure	Moderator measure	Results	Quality appraisal grade
													ED risk prone adolescents engage in certain SM activities which maintains or exacerbates their disorder	
33.	Prichard et al (2020)	Australia<br><br>High-income economy	Experimental	N (% F)<br>Setting<br>Age (mean +- SD)<br>BMI<br><br>Ethnicity<br><br><br>Sexual orientation<br>ED prevalence	108 (100% F)<br>University<br>17-25 (20.24+-1.86)<br>23.02 (H)<br>White 64.8%, Asian 27.8%, Indian 3.7%, 2.8% other<br>NR<br>NR	Investigated the effect of exposure to fitspiration images via IG on mood, BID and exercise behaviour in young women	Sociocultural theory<br><br>Social Comparison Theory	IG	Experimental exposure to fitspiration imagery: randomly assigned to fitspiration or travel images followed by exercise or rest<br><br>SM usage: frequency and time spent/day	Mood & BID: Visual analogue scales<br><br>Exercise behaviour: distance travelled on treadmill and perceived exertion in 10-minute time frame	N/A	N/A	Exposure to fitspiration resulted in significantly higher negative mood & BID relative to control travel images<br>There was no difference in exercise behaviour between the two groups: fitspiration did not motivate exercise (main	Moderate

#	Author	Country & World Bank Classification	Study Design	Participant characteristics		Objectives	Underlying theoretical framework	Social media platform	Exposure measure (Social media use)	Outcome measure (BI/ED symptomatology)	Mediator measure	Moderator measure	Results	Quality appraisal grade
													'goal' of fitspiration)	
34.	Rodgers et al (2020)	Australia<br><br>High-income economy	CS	N (% F)<br>Setting<br>Age (mean +-SD)<br>BMI<br>Ethnicity<br>Sexual orientation<br>ED prevalence	681 (49% F)<br>Secondary school<br>NR (12.76 +-0.74)<br>NR<br>NR<br>NR<br>NR	Investigated the association between SM use and BI concerns and body changing behaviours<br>Explored internalization of thin/fit ideal and upward appearance comparisons<br>Assessed whether this may be moderated by negative affect (depression & self-esteem)	Biopsychosocial model	Multiple: SC, IG, FB, YT Twitter, Tumblr, Pintere-st	SM usage: self-report questionnaire	BID: subscale from the Eating Disorders Examination Questionnaire (EDE-Q)<br><br>Muscle building behaviours: The Body Change Inventory	Internalization of appearance ideals: Muscular subscale of the Sociocultural Attitudes towards appearance questionnaire	Depression: Center for Epidemiologic Studies Depression Scale revised version for adolescents<br><br>Self Esteem: Established single measure<br><br>Gender<br><br>BMI: Self-reported height and weight	SM use was significantly associated with BID, dietary restraint and muscle building behaviours in girls and boys (novel outcome for girls)<br>Self-esteem and depressive symptoms → SM ideal internalization & higher appearance comparison (support for moderator)<br>Higher BMI was directly associated with	Moderate

#	Author	Country & World Bank Classification	Study Design	Participant characteristics		Objectives	Underlying theoretical framework	Social media platform	Exposure measure (Social media use)	Outcome measure (BI/ED symptomatology)	Mediator measure	Moderator measure	Results	Quality appraisal grade
													BID (moderator)<br>SM ideal internalization & higher appearance comparison → BID (support for mediators)<br>Gender only moderated the relationship between SM & muscular ideal internalization – only significant for boys<br>Implies that vulnerabilities and high BMI predispose an individual to the detrimental effects of SM and BID (mediated through ideal internalization &	

#	Author	Country & World Bank Classification	Study Design	Participant characteristics		Objectives	Underlying theoretical framework	Social media platform	Exposure measure (Social media use)	Outcome measure (BI/ED symptomatology)	Mediator measure	Moderator measure	Results	Quality appraisal grade
													appearance comparisons)	
35.	Scully et al (2020)	Ireland<br><br>High-income economy	CS	N (% F)<br>Setting<br>Age (mean +- SD)<br>BMI<br>Ethnicity<br>Sexual orientation<br>ED prevalence	210 (100% F)<br>Secondary school<br>12-17 (15.16 +- 1.17)<br>NR<br>NR<br>NR<br>NR	Examined whether appearance related SM activity is associated with BID amongst adolescent girls<br>Explored whether social comparisons and thin-ideal internalization mediated this relationship	Sociocultural model<br><br>Social comparison theory<br><br>Objectification theory	FB	Appearance related SM exposure: 8-item photo subscale	BID: BID subscale of the ED inventory	SM comparisons: Items devised by Fardouly and Vartanian (2015)<br><br>Internalisation of the thin ideal: The thin-ideal internalisation scale	N/A	Demonstrated a significant positive relationship between appearance-based SM and body dissatisfaction via the mediating pathways of social comparison and internalization of the thin ideal<br>Time spent engaging in appearance related FB content was not directly associated with BID- only via the mediating	High

#	Author	Country & World Bank Classification	Study Design	Participant characteristics		Objectives	Underlying theoretical framework	Social media platform	Exposure measure (Social media use)	Outcome measure (BI/ED symptomatology)	Mediator measure	Moderator measure	Results	Quality appraisal grade
													pathways	
36.	Tiggemann and Andenberg (2020)	US<br><br>High-income economy	Experimental	N (% F)<br>Setting<br>Age (mean +- SD)<br>BMI<br>Ethnicity<br><br><br><br><br><br>Sexual orientation<br>ED prevalence	300 (0% F)<br>Online<br>18-30 (24.94 +- 2.96)<br>26.01 (OW)<br>White 61.3%, African American 12.7%, Latino 11.7%, Asian 10.3%, Native American 2.0%, other 2%<br>NR<br>NR	Investigated the effects of exposure to idealised male fitspiration images on men's BI	Sociocultural theory<br><br>Social comparison theory	IG	SM usage: self-report questionnaire<br><br>Experimental exposure to bare-chested 'fitspiration' IG images, fashion images or control travel images	BI and facial satisfaction: validated visual analogue scales	Appearance comparison: The State Appearance Comparison Scale<br><br>Muscular Ideal Internalization: The Muscular Subscale of the Sociocultural Attitudes towards Appearance Questionnaire-4 revised	N/A	Exposure to bare-chested fitspiration images → greater BID in contrast to fashion images and travel images<br>Social comparison and muscular ideal internalization were not significant mediators of this relationship, demonstrating that other mechanisms may be at play for adult males	High
37.	Verrastro et al (2020)	Italy<br><br>High-	CS	N (% F)<br>Setting<br>Age	621 (61% F)<br>Secondary school	Investigated the relationship between IG use	Impression management theory	IG	IG usage: self-reported questionnaire	BI & anxiety:<br><br>Sociocultural	Photo investment: editing	N/A	Adolescent girls and boys who edit photos and	High

#	Author	Country & World Bank Classification	Study Design	Participant characteristics		Objectives	Underlying theoretical framework	Social media platform	Exposure measure (Social media use)	Outcome measure (BI/ED symptomatology)	Mediator measure	Moderator measure	Results	Quality appraisal grade
		income economy		(mean +- SD)<br>BMI<br>Ethnicity<br>Sexual orientation<br>ED prevalence	13-21 (15.90 +- 1.55)<br>NR<br>NR<br>NR<br>NR	and photo editing, internalization of 'beauty' standards, pressure to adhere to these & BI anxieties	Objectification theory			aptitudes toward appearance questionnaire<br><br>Fear of negative appearance evaluation scale<br><br>Physical appearance state and trait anxiety scale	photos and posting online		post these online have greater internalized stereotypes of beauty, feel greater pressure to adhere to this, experience higher BI related anxiety and report higher fear of negative evaluation from others<br>Females had higher levels of BI related anxiety and internalized the beauty ideal more than male counterparts (gender as a slight moderator)<br>Fear of negative evaluation from others &	

#	Author	Country & World Bank Classification	Study Design	Participant characteristics		Objectives	Underlying theoretical framework	Social media platform	Exposure measure (Social media use)	Outcome measure (BI/ED symptomatology)	Mediator measure	Moderator measure	Results	Quality appraisal grade
													pressure to uphold the IG ideal was equal for both genders	
38.	Wick and Keel (2020)	US<br><br>High-income economy	Mixed methods: CS & experimental (2 stage design)	Stage 1:<br>N (% F)<br>Setting<br>Age (mean +- SD)<br>BMI<br>Ethnicity<br><br><br><br>Sexual orientation<br>ED prevalence<br><br><br>Stage 2:<br>N (% F)<br>Setting<br>Age	Cross sectional<br>2,485 (76% F)<br>University<br>(19.01 +- 1.80)<br>NR<br>77% White, 10% Black, 4% Asian, 2% Other, 23% Hispanic<br>NR<br>NR<br><br><br>Experimental<br>89 (93% F)<br>University<br>(18.71 +- 0.97)<br>22.64<br>91% White, 8% Black, 6%	Investigated the association between posting edited photos on IG and DE, anxiety & depressive symptoms amongst university males and females	Impression management theory<br><br>Gratification's theory	IG	Stage 1. self-reported questionnaire on SM usage<br><br>Stage 2. Participants took a full body photo and randomised to one of four conditions:<br>1) edit and post photo on IG 2) edit but not post 3) post unedited version 4) control	DE: EAT-26 scale<br><br>Anxiety: Penn State Worry questionnaire<br><br>Depressive symptoms: Patient Health Questionnaire-9	Modifying photos: self-reported and as part of experiment<br><br>Problematic IG use: self-reported comparison, addiction, and investment in peer feedback	Gender<br><br>Ethnicity	Stage 1: CS<br>Direct link between posting edited photos and ED risk factors and anxiety but no relationship with depressive symptoms<br>Posting photos was more common for females and for White and Asian participants<br><br>Stage 2: Experimental<br>Participants assigned to the posting an edited	Moderate

#	Author	Country & World Bank Classification	Study Design	Participant characteristics		Objectives	Underlying theoretical framework	Social media platform	Exposure measure (Social media use)	Outcome measure (BI/ED symptomatology)	Mediator measure	Moderator measure	Results	Quality appraisal grade
				(mean +- SD)<br>BMI<br>Ethnicity<br><br><br>Sexual orientation<br>ED prevalence	Asian, 1% other, 24% Hispanic<br>NR<br>NR								photo of themselves condition had significant increases in weight/shape concerns with a large effect size. This heightened anxiety and increased urges to exercise and restrict food intake<br>Just 5 minutes of editing a photo led to sadness and increased weight/shape concerns	
39.	Wilksch et al (2020)	Australia<br><br>High-income economy	CS	N (% F)<br>Setting<br>Age (mean +- SD)<br>BMI<br>Ethnicity	996 (53.6% F)<br>Secondary school<br>NR (13.08 +- 0.60)<br>18.94<br>NR	Investigated the relationship between SM usage and rates of DE eating in young adolescent boys	Sociocultural theory	Multiple: FB, IG, SC, Tumblr	SM use: self-report questionnaire	DE cognitions: EDE-Q<br><br>DE behaviours: Project EAT assessment	Time spent on SM and number of platforms visited	Gender	DE cognitions and behaviours and SM usage were significantly associated, with greater effect sizes for IG, SC	Low

#	Author	Country & World Bank Classification	Study Design	Participant characteristics		Objectives	Underlying theoretical framework	Social media platform	Exposure measure (Social media use)	Outcome measure (BI/ED symptomatology)	Mediator measure	Moderator measure	Results	Quality appraisal grade
				Sexual orientation<br>ED prevalence	NR<br>NR but DE behaviours reported by 51.7% girls and 45% boys	and girls							& FB (image-oriented platforms).<br>DE behaviours included strict exercise and meal skipping and were reported by 51.7% girls and 45% boys<br>Time spent on SM & number of SM sites visited were significant mediators of this relationship<br>Girls posted more photos of people and food and displayed higher levels of DE behaviours	
**40.**	Yang et al (2020)	Singapore<br><br>High-income	CS	N (% F)<br>Setting<br>Age (mean +-	100 (100% F)<br>Community<br>13-18 (15.07 +- 1.33)	Investigated the relationship between SM use and BI	Cognitive affective model	MultipleFB, IG, SC, Twitter	SM use: modified version of the media and	BI: Body esteem scale for adolescents and adults	Internalisation of the ideal: Cognitive	N/A	Excessive SM use (+3 hour daily) negatively impacts BE in	High

#	Author	Country & World Bank Classification	Study Design	Participant characteristics		Objectives	Underlying theoretical framework	Social media platform	Exposure measure (Social media use)	Outcome measure (BI/ED symptomatology)	Mediator measure	Moderator measure	Results	Quality appraisal grade
		economy		SD)<br>BMI<br>Ethnicity<br><br><br>Sexual orientation<br>ED prevalence	NR<br>60% Chinese, 18% Malay, 9% Indian. 13% Other<br>NR<br>NR	Explored whether internalization of the idea, appearance comparison and social appearance anxiety mediated this relationship			technology usage attitudes scale	Weight locus of control: 16-item dieting beliefs scale to assess participants control beliefs about their weight	internalization subscale from the Sociocultural Attitudes Towards Appearance Scale<br><br>Appearance comparisons: 10-item adapted version of the Physical Appearance Scale<br><br>Appearance anxiety: 16-item Social Appearance Anxiety Scale		female adolescents<br>This relationship was significantly mediated by internalisation of the thin/fit ideal & upward appearance comparisons<br>Found that both online and real-life comparisons mediated the relationship between excessive SM use and BE<br>Directionality cannot be assumed	
**41.**	Yao et al (2020)	China<br><br>Upper-	CS	N (% F)<br>Setting<br>Age	567 (100% F)<br>University<br>17-23 (19.97	Investigated the relationship between BI	Objectification theory	General	SM usage: self-report questionnaire	Restrained eating: Restrained	Body shame: Body shame	Body appreciation: The Body	BI comparisons and body shame mediated the	High

#	Author	Country & World Bank Classification	Study Design	Participant characteristics		Objectives	Underlying theoretical framework	Social media platform	Exposure measure (Social media use)	Outcome measure (BI/ED symptomatology)	Mediator measure	Moderator measure	Results	Quality appraisal grade
		middle-income economy		(mean +- SD)<br>BMI<br>Ethnicity<br>Sexual orientation<br>ED prevalence	+- 1.37)<br>19.83<br>NR<br>NR<br>NR	comparisons on SM, body shame and restrained eating amongst Chinese university students<br>Explored whether body appreciation and BMI moderated this relationship	Social comparison theory			eating subscale of the Dutch Eating Behaviour Questionnaire (DEBQ)	subscale of the Chinese version of the Objectified Body Consciousness Scale	Appreciation Scale-2<br><br>BMI	relationship between SM usage and restrained eating<br>SM usage → BI comparisons → Body shame → Restrained eating<br>Body appreciation* buffered the relationship between BS and restrained eating<br>The relationship between BS and RE was stronger amongst women with lower BMI but BI comparisons and RE was stronger for women with a higher BMI (BMI as a moderator)	

#	Author	Country & World Bank Classification	Study Design	Participant characteristics		Objectives	Underlying theoretical framework	Social media platform	Exposure measure (Social media use)	Outcome measure (BI/ED symptomatology)	Mediator measure	Moderator measure	Results	Quality appraisal grade
42.	Baminiwatta et al (2021)	Sri Lanka<br><br>Lower-middle income economy	CS	N (% F)<br>Setting<br><br>Age (mean +- SD)<br>BMI<br>Ethnicity<br>Sexual orientation<br>ED prevalence	478 (46.3%)<br>Secondary school<br>13-19 (NR)<br>NR<br>NR<br>NR<br>NR<br>NR	Investigated the relationship between SM behaviours & BID amongst adolescents	Impression management theory	General	SM usage: time spent on SM and frequency of selfie posting, selfie-taking & photo manipulation using ordinal scale developed by McLean et al (2015)	BID: Self-administered BI satisfaction scale	SM activities: selfie-taking, selfie-posting, and photo manipulation	Gender<br><br>BMI	Taking 'selfies' was associated with BID for the whole sample whilst photo manipulation was significantly associated with BID amongst girls only<br>SM activities were comparable to young women in Australia<br>BMI was directly associated with BID	Low
43.	Dignard and Jarry (2021)	Canada<br><br>High-income economy	Experimental	N (% F)<br>Setting<br>Age (mean +- SD)<br>BMI<br>Ethnicity	340 (100%)<br>University<br>17-43 (20.61 +- 2.64)<br>23.35kg<br>77.7% White, 9.3% Arab,	Experimentally compared the effects of fitspiration and thinspiration on women's BI	Sociocultural theory<br><br>Social comparison theory	IG	Experimental exposure to either thinspiration, fitspiration or control travel images on IG	BI: 10-item Body Appreciation Scale	State appearance comparison: Items validated by Tiggemann and McGill	State body satisfaction: 10-item Body Appreciation scale	Viewing fitspiration and thinspiration was equally associated with BID relative to viewing control	Moderate

#	Author	Country & World Bank Classification	Study Design	Participant characteristics		Objectives	Underlying theoretical framework	Social media platform	Exposure measure (Social media use)	Outcome measure (BI/ED symptomatology)	Mediator measure	Moderator measure	Results	Quality appraisal grade
				Sexual orientation<br>ED prevalence	6.6% African, 5.2% South Asian, 2.7% East Asian<br>NR<br>NR				SM usage: SM platforms used, frequency, specific activities		(2004)	Ethnicity	travel images<br>This was mediated by engagement appearance comparisons, which was higher for the Fitspiration group<br>Higher state positive BI only inhibited appearance comparison with thinspiration and not fitspiration – protective against emaciated thinspiration images but NOT fitspiration<br>Non-white – only fitspiration → body dissatisfaction	

#	Author	Country & World Bank Classification	Study Design	Participant characteristics		Objectives	Underlying theoretical framework	Social media platform	Exposure measure (Social media use)	Outcome measure (BI/ED symptomatology)	Mediator measure	Moderator measure	Results	Quality appraisal grade
													but this was NOT mediated by appearance comparison	
44.	Ding and Xu (2021)	China<br><br>Upper-middle-income economy	CS	N (% F)<br>Setting<br>Age (mean +- SD)<br>BMI<br>Ethnicity<br>Sexual orientation<br>ED prevalence	551 (51.3%)<br>Secondary school<br>15-17 (16.11)<br>NR<br>NR<br>NR<br>NR	Investigated the relationship between appearance-based comparisons on SM, anxiety and emotional eating<br>Explored anxiety as a mediator	Sociocultural theory<br><br>Social identity theory	Chinese micro-blogs	SM usage: appearance-based SM comparisons using revised scale by Fardouly and Vartanian (2015)	Emotional eating: Emotional Eating Scale (EES-C scale) adapted for use in children and adolescents	Anxiety: anxiety subscale of the Depression Anxiety Stress Scale (DASS)	Sociocultural pressure: subscale from the Sociocultural Attitudes towards appearance questionnaire<br><br>Gender	Social comparisons on SM → emotional eating which was fully mediated by anxiety amongst both genders<br>Females were slightly more sensitive to appearance comparisons on SM and showed more emotional eating tendencies in contrast to male counterparts (gender partly moderated)	Moderate
45.	Jarman et al (2021)	Australia	CS	N (% F)<br>Setting	1,579 (55.6% F)	Investigated whether	Tripartite influence	General	SM usage: intensity and	BI: Modified version of the	Thin and muscular	Gender	Only higher appearance	Moderate

#	Author	Country & World Bank Classification	Study Design	Participant characteristics		Objectives	Underlying theoretical framework	Social media platform	Exposure measure (Social media use)	Outcome measure (BI/ED symptomatology)	Mediator measure	Moderator measure	Results	Quality appraisal grade
		High-income economy		Age (mean +- SD)<br>BMI<br>Ethnicity<br><br>Sexual orientation<br>ED prevalence	Secondary school<br>11-17 (13.45 +- 1.15)<br>NR<br>85.55% White, Asian (8.27%)<br>NR<br>NR	appearance ideal (thin & muscular) internalization and social appearance comparisons mediated relationships between SM engagement (intensity & appearance focused) and BI and wellbeing	model		appearance focused SM use	Body Shape Satisfaction scale<br><br>Two items from the Weight and Shape subscales of the Eating Disorder Examination Questionnaire (EDE-Q) to measure overvaluation of weight and shape<br><br>The Appearance Esteem subscale of the Body Esteem scale<br><br>Wellbeing: the 5-item	ideal internalization:<br>The Thin/Low Body Fat subscale of the Sociocultural Attitudes Towards Appearance Questionnaire-4 (SATAQ-Internalization of the muscular-ideal was assessed using the Muscular subscale of the SATAQ-4R-Male<br><br>Social and appearance		focused SM use was directly associated with BID and lower wellbeing<br>Higher appearance ideal internalization and appearance comparisons mediated the relationship between higher SM engagement and BID and lower wellbeing, which was strongest for comparisons<br>SM intensity did not directly → BID and low wellbeing (only via the mediating pathways)<br>Gender was not	

#	Author	Country & World Bank Classification	Study Design	Participant characteristics		Objectives	Underlying theoretical framework	Social media platform	Exposure measure (Social media use)	Outcome measure (BI/ED symptomatology)	Mediator measure	Moderator measure	Results	Quality appraisal grade
										satisfaction with life scale	comparison: Frequency of comparisons on SM<br>5 items from the Upward Physical Appearance Comparison Scale		a moderator although boys were more likely to internalise the muscular ideal	
46.	Jiotsa et al (2021)	France<br><br>High-income economy	CS	N (% F)<br>Setting<br>Age (mean +- SD)<br>BMI<br>Ethnicity<br>Sexual orientation<br>ED prevalence	1331 (97.7% F)<br>Community<br>15-35 (24.4 +- 4.2)<br>22.3<br>NR<br>NR<br>193 patients (14.5%)	Investigated the association between how often one compared their physical appearance to that of people followed on SM and one's BID and drive for thinness and ED prevalence	Sociocultural theory<br><br>Social comparison theory<br><br>Gratification's theory	Multiple	SM usage: platform, frequency, time spent, frequency of comparisons, frequency of selfie posting	BI: Eating disorder Inventory (EDI-2) scale translated and adapted into French – includes Drive for Thinness and Body Dissatisfaction subscales<br><br>ED screening: Sick-Control-One-Stone-Fat-	N/A	BMI	ED or at-risk ED subjects (based on SCOFF score) demonstrated higher rates of BID and greater drive for thinness<br>Significant association between frequency of comparing one's own physical appearance to that of people	Moderate

#	Author	Country & World Bank Classification	Study Design	Participant characteristics		Objectives	Underlying theoretical framework	Social media platform	Exposure measure (Social media use)	Outcome measure (BI/ED symptomatology)	Mediator measure	Moderator measure	Results	Quality appraisal grade
										Food (SCOFF) questionnaires			followed on SM and BD and drive for thinness<br><br>Plausible that this is bidirectional i.e., appearance comparisons on SM → BID and drive for thinness OR that state BID and drive for thinness → greater appearance comparisons on SM<br>BMI was not a moderator	
47.	Limniou et al (2021)	UK<br><br>High-income economy	Mixed methods: experimental and CS	N (% F)<br>Setting<br>Age (mean +- SD)<br>BMI	109 (86.20% F)<br>University<br>18-50 (20 +- 3.3)<br>NR	Investigated the effects of viewing fitspiration photos on BI and fit-ideal	Tripartite influence model	IG	Experimental exposure to fitspiration on IG	BI: 5-item body satisfaction scale<br><br>Mood: 5-item mood	Fit ideal internalisation: 11-item Fit Ideal Internalization Scale	Gender<br><br>Age	Exposure to fitspiration produced a significant reduction in state self-esteem,	Low

#	Author	Country & World Bank Classification	Study Design	Participant characteristics		Objectives	Underlying theoretical framework	Social media platform	Exposure measure (Social media use)	Outcome measure (BI/ED symptomatology)	Mediator measure	Moderator measure	Results	Quality appraisal grade
				Ethnicity<br>Sexual orientation<br>ED prevalence	NR<br>NR<br>NR	internalisation (measuring SE, BS, mood satisfaction and fit-ideal internalisation)				satisfaction scale<br><br>SE: 5-item self-esteem scale			mood satisfaction and fit-ideal internalisation and had no significant impact on body satisfaction<br>Fit-ideal internalisation decreased after viewing fitspiration images (other mechanisms must be at play)<br>Age was not a moderator<br>Gender was a moderator: males internalized the fit ideal more but women demonstrated lower mood satisfaction	

#	Author	Country & World Bank Classification	Study Design	Participant characteristics		Objectives	Underlying theoretical framework	Social media platform	Exposure measure (Social media use)	Outcome measure (BI/ED symptomatology)	Mediator measure	Moderator measure	Results	Quality appraisal grade
													Specific IG activities e.g. investment in likes, was negatively associated with self-esteem, mood and BS<br>Higher IG frequency use and posting was related to higher state self-esteem	
48.	Mahon and Hevey (2021)	Ireland<br><br>High-income economy	Qualitative focus groups	N (% F)<br>Setting<br>Age (mean +- SD)<br>BMI<br>Ethnicity<br>Sexual orientation<br>ED prevalence	29 (79.3% F)<br>Secondary school<br>15-16 (15.31 +- 0.47)<br>NR<br>NR<br>NR<br>NR	Explored adolescent's experiences of SM on BI and any coping mechanisms employed	Tripartite model<br><br>Impression management theory	General	General SM use explored during focus groups	BI	Comparison with peers<br><br>Investment in likes	Gender<br><br>SM literacy<br><br>Protective filtering	Demonstrated that despite considerable awareness to filter out damaging BI content, its presence on SM sites is ubiquitous. Students reported comparing to others, trying to	High

#	Author	Country & World Bank Classification	Study Design	Participant characteristics		Objectives	Underlying theoretical framework	Social media platform	Exposure measure (Social media use)	Outcome measure (BI/ED symptomatology)	Mediator measure	Moderator measure	Results	Quality appraisal grade
													look like everyone else, ‘hating’ photos of themselves, picking out flaws and self-blame for failing to adhere to the ideal.<br>Discussed exposure to advertising for weight loss on SM platforms<br>Participants were critical of BP movements and claimed that skinnier people/those closer to the ideal receive more rewards (comments and likes)<br>Girls were more aware of editing,	

#	Author	Country & World Bank Classification	Study Design	Participant characteristics		Objectives	Underlying theoretical framework	Social media platform	Exposure measure (Social media use)	Outcome measure (BI/ED symptomatology)	Mediator measure	Moderator measure	Results	Quality appraisal grade
													placed huge emphasis on likes, and felt very strongly about comparisons vs boys who showed greater body esteem, saw the muscular ideal as motivating (functionality), and were less aware of editing/manipulation of male bodies	
**49.**	Stein et al (2021)	Germany<br><br>High-income economy	CS	N (% F)<br>Setting<br>Age (mean +- SD)<br>BMI<br>Ethnicity<br>Sexual	228 (75% F)<br>Online<br>18-34 years (22.5 +- 3.04)<br>NR<br>NR<br>NR	Investigated SM use (IG) as a potential cultivation system for young adult's BI	Cultivation theory	IG	SM usage: self-reported weekly time spent on IG and activities i.e., browsing public content	BI: Body Esteem Scale for Adolescents and Adults<br><br>Dietary restraint: The EAT-26	IG activities	Gender	Time on IG did not significantly predict any outcomes of interest<br>Strong tendency to browse IG predicted stricter	High

#	Author	Country & World Bank Classification	Study Design	Participant characteristics		Objectives	Underlying theoretical framework	Social media platform	Exposure measure (Social media use)	Outcome measure (BI/ED symptomatology)	Mediator measure	Moderator measure	Results	Quality appraisal grade
				orientation<br>ED prevalence									views on the weight of strangers (particularly for women) and increased risk for DE for both males and females<br>Females were more likely to overestimate frequency of BS in the general population and hold unfavourable views of their own body<br>IG is a meaningful cultivation system for body-related attitudes and behaviours amongst young adults	

#	Author	Country & World Bank Classification	Study Design	Participant characteristics		Objectives	Underlying theoretical framework	Social media platform	Exposure measure (Social media use)	Outcome measure (BI/ED symptomatology)	Mediator measure	Moderator measure	Results	Quality appraisal grade
50.	Vall-Roque et al (2021)	Spain<br><br>High-income economy	CS	N (% F)<br>Setting<br>Age (mean +- SD)<br>BMI<br>Ethnicity<br>Sexual orientation<br>ED prevalence	2601 (100% F)<br>Community<br>14-35 (24.05)<br>23.79kg (H)<br>NR<br>NR<br>NR	Aimed to determine the impact of COVID-19 lockdown on SNS and its association with body image disturbances and low SE	Sociocultural theory	Multiple: IG, YouTube, TikTok, Twitter and FB	SM usage: self-report SM items assessing frequency of SM use pre and during lockdown and types of accounts followed on IG	BI: Drive for thinness and BID subscales of the ED inventory<br><br>Self-esteem: Rosenberg self-esteem scale	N/A	N/A	Statistically significant association between frequency of all studied SNSs during lockdown, following appearance focused accounts & subsequent drive for thinness, low self-esteem and BID amongst generation z participants (14-24)<br>This did not lead to BID for the older age group >24	Moderate

NR = not reported

N/A = not applicable
