# Supplementary material for: The social media diet: A scoping review to investigate the association between social media, body image and eating disorders amongst young people
Source: PLOS Glob Public Health. 2023 Mar 22;3(3):e0001091. doi: 10.1371/journal.pgph.0001091 (PMC10032524; doi:10.1371/journal.pgph.0001091)
Supplement: S2 Table — (PDF) [file pgph.0001091.s003.pdf]

S3 Table. Additional theories outside of Rodgers' framework

THEORY	EXPLANATION
Social learning theory	Individuals observe and imitate the behaviours of others, particularly when that behaviour is rewarded. This is hypothesized to play a role in the development of body image (BI) attitudes and disordered eating (DE) behaviours e.g., dieting
Social comparison theory	Self-evaluation is derived from comparisons to similar others. Comparisons can be upward (comparing to someone deemed superior) or downward (comparing to someone deemed inferior)
Cultivation theory	Immersion in social media shapes attitudes, perceptions and behaviours based on what is perceived as the norm
Cognitive affective model	Hypothesises that the internalisation of the thin ideal leads to social comparison and subsequent poor body esteem outcomes
Tripartite influence model	Postulates that the media (social or mass), parents and peers contribute to the development of appearance ideals, social comparison and subsequent BI or DE outcomes
Biopsychosocial model	Brings together both biological and sociocultural theories of BI and eating disorders (ED). Indicates that self-esteem, depressive state, and SM use → internalisation of the thin/muscular ideal & upward appearance comparison → body image dissatisfaction (BID) & dietary

	restraint & muscle building behaviours
Dual pathway model	Postulates that social environmental pressures to be thin (media, family, and peers) leads to thin-ideal internalisation. Where an individual is unable to actualise this ideal, this leads to BID. BID is hypothesised to lead to bulimia symptomatology via two pathways: dietary restraint (& subsequent binging /purging) and negative emotionality (& subsequent binging / purging as a distraction)
