# Supplementary material for: The social media diet: A scoping review to investigate the association between social media, body image and eating disorders amongst young people
Source: PLOS Glob Public Health. 2023 Mar 22;3(3):e0001091. doi: 10.1371/journal.pgph.0001091 (PMC10032524; doi:10.1371/journal.pgph.0001091)
Supplement: S3 Table — (PDF) [file pgph.0001091.s004.pdf]

S4 Table. The Joanna Briggs Institute (JBI) checklist for analytical cross-sectional studies

Utilised for: cross-sectional, ecological momentary assessment, mixed methods and longitudinal observational studies

Study	Were the criteria for inclusion in the sample clearly defined?	Were the study subjects and setting described in detail?	Was the exposure measured in a valid and reliable way?	Were objective, standard criteria used for measurement of the outcome(s)?	Were confounding factors identified?	Were strategies to deal with confounding factors stated?	Was appropriate statistical analysis used?	Total score (/7)	Grade
1. Baminiwatta et al (2021)	NO	N0	PARTLY	YES	PARTLY	NO	UNCLEAR	1.5/7	LOW
2. Ding and Xu (2021)	PARTLY	YES	YES	YES	NO	NO	YES	4.5/7	MODERATE
3. Jarman et al (2021)	YES	YES	YES	YES	NO	NO	YES	5/7	MODERATE
4. Jiotsa et al (2021)	YES	PARTLY	PARTLY	YES	YES	NO	YES	5/7	MODERATE
5. Stein et al (2021)	YES	NO	YES	YES	YES	YES	YES	6/7	HIGH
6. Vall-Roque (2021)	YES	NO	YES	YES	NO	NO	YES	4/7	MODERATE
7. Bennett et al (2020)	YES	YES	YES	YES	NO	NO	YES	5/7	MODERATE
8. Cavazos-Rehg et	YES	PARTLY	YES	YES	NO	NO	YES	4.5/7	MODERATE

	al (2020)									
9.	Choukas-Bradley et al (2020)	NO	PARTLY	YES	YES	NO	NO	YES	3.5/7	MODERATE
10.	Fitzsimmons-Craft (2020)	YES	YES	PARTLY	YES	NO	NO	YES	4.5/7	MODERATE
11.	Gioia (2020)	NO	NO	YES	YES	NO	NO	YES	3/7	LOW
12.	Krug et al (2020)	PARTLY	YES	YES	YES	PARTLY	PARTLY	YES	5.5/7	HIGH
13.	Lonegan et al (2020)	YES	YES	YES	YES	PARTLY	PARTLY	YES	6/7	HIGH
14.	Rodgers et al (2020)	PARTLY	YES	PARTLY	YES	PARTLY	PARTLY	YES	5/7	MODERATE
15.	Scully et al (2020)	NO	PARTLY	YES	YES	YES	YES	YES	5.5/7	HIGH
16.	Verrastro et al (2020)	YES	PARTLY	YES	YES	YES	YES	YES	6.5/7	HIGH
17.	Wilksch et al (2020)	NO	PARTLY	PARTLY	YES	UNCLEAR	UNCLEAR	YES	3/7	LOW
18.	Yang et al (2020)	NO	PARTLY	YES	YES	YES	YES	YES	5.5/7	HIGH
19.	Yao et al (2020)	NO	PARTLY	YES	YES	YES	YES	YES	5.5/7	HIGH
20.	Aparicio-Martinez et al (2019)	YES	PARTLY	PARTLY	YES	UNCLEAR	UNCLEAR	YES	4/7	MODERATE
21.	Chang et al (2019)	PARTLY	YES	YES	YES	UNCLEAR	UNCLEAR	YES	4.5/7	MODERATE
22.	De Vries et al (2019)	NO	YES	YES	YES	YES	YES	YES	6/7	HIGH

23.	Lonergan et al (2019)	YES	PARTLY	YES	YES	YES	YES	YES	6.5/7	HIGH
24.	Marengo et al (2018)	NO	PARTLY	PARTLY	YES	YES	YES	YES	5/7	MODERATE
25.	Raggatt et al (2018)	YES	YES	PARTY	YES	NO	NO	YES	4.5/7	MODERATE
26.	Saunders and Eaton (2018)	PARTLY	PARTLY	PARTLY	YES	NO	NO	YES	3.5/7	MODERATE
27.	Ahadzadeh et al (2017)	YES	YES	PARTLY	YES	UNCLEAR	UNCLEAR	YES	4.5/7	MODERATE
28.	Cohen et al (2017)	NO	YES	YES	YES	NO	NO	UNCLEAR	3.5/7	MODERATE
29.	Howard et al (2017)	YES	YES	YES	YES	NO	NO	YES	5/7	MODERATE
30.	Kaewpradub et al (2017)	NO	NO	PARTLY	YES	YES	YES	YES	4.5/7	MODERATE
31.	Rousseau et al (2017)	NO	NO	YES	YES	YES	YES	YES	5/7	MODERATE
32.	Santarossa and Woodruff(2017)	NO	YES	YES	YES	UNCLEAR	UNCLEAR	YES	4/7	MODERATE
33.	Turner and Lefevre (2017)	PARTLY	YES	PARTLY	YES	YES	YES	YES	5.5/7	HIGH
34.	Murray et al (2016)	NO	YES	YES	YES	YES	YES	YES	6/7	HIGH
35.	Puccio et al (2016)	NO	PARTLY	YES	YES	YES	YES	YES	5.5/7	HIGH

36. Tan et al (2016)	YES	YES	PARTLY	YES	NO	NO	YES	4.5/7	MODERATE
