# Supplementary material for: The social media diet: A scoping review to investigate the association between social media, body image and eating disorders amongst young people
Source: PLOS Glob Public Health. 2023 Mar 22;3(3):e0001091. doi: 10.1371/journal.pgph.0001091 (PMC10032524; doi:10.1371/journal.pgph.0001091)
Supplement: S4 Table — (PDF) [file pgph.0001091.s005.pdf]

S5 Table. Adapted version of the Joanna Briggs Institute tool for quasi-experimental studies

Utilised for: Experimental and mixed methods experimental studies

Study	Is it clear what is the 'exposure' and what is the 'outcome'?	Were participants included in comparisons similar?	Were both conditions the same except for the exposure?	Was there a control group?	Were there multiple measurements of the outcome both pre and post the exposure?	Was follow up complete and if not, were differences in follow up described adequately?	Were outcomes of participants included in comparisons measured in the same way?	Were outcomes measured in a reliable way?	Was appropriate statistical analysis used?	Total (/9)	Grade
1. Disgnard and Jarry (2021)	YES	YES	YES	YES	UNCLEAR	UNCLEAR	YES	PARTLY	YES	6.5/9	MODERATE
2. Limniou et al (2021)	YES	UNCLEAR	UNCLEAR	NO	NO	NO	N/A	PARTLY	YES	2.5/9	LOW
3. Prichard et al (2020)	YES	UNCLEAR	YES	YES	UNCLEAR	UNCLEAR	YES	PARTLY	YES	5.5/9	MODERATE
4. Tiggemann and Andenberg (2020)	YES	YES	UNCLEAR	YES	YES	YES	YES	PARTLY	YES	7.5/9	HIGH
5. Wick and Keel (2020)	YES	UNCLEAR	UNCLEAR	YES	YES	YES	YES	PARTLY	YES	6.5/9	MODERATE
6. Kleemans et al (2018)	YES	UNCLEAR	UNCLEAR	YES	NO	NO	YES	PARTLY	YES	5.5/9	MODERATE
7. Tiggemann et al	YES	UNCLEAR	UNCLEAR	NO	YES	NO	YES	PARTLY	YES	4.5/9	MODERATE

	(2018)										
8.	Brown and Tiggemann (2016)	YES	YES	UNCLEAR	YES	YES	NO	YES	PARTLY	YES	6.5/9	MODERATE
9.	Kim and Park (2016)	YES	UNCLEAR	UNCLEAR	NO	YES	NO	YES	PARTLY	YES	4.5/9	MODERATE
