# Supplementary material for: The social media diet: A scoping review to investigate the association between social media, body image and eating disorders amongst young people
Source: PLOS Glob Public Health. 2023 Mar 22;3(3):e0001091. doi: 10.1371/journal.pgph.0001091 (PMC10032524; doi:10.1371/journal.pgph.0001091)
Supplement: S5 Table — (PDF) [file pgph.0001091.s006.pdf]

S6 Table. The Critical Appraisal Skills Checklist (CASP) utilised for qualitative studies

Study	Was there a clear statement of the aims of the research?	Is a qualitative methodology appropriate?	Was the research design appropriate to address aims?	Was the recruitment strategy appropriate to aims?	Was the methodology adequately justified?	Has the relationship between researcher & participants been considered?	Have ethical issues been considered?	Was the data analysis sufficiently rigorous?	Is there a clear statement of findings?	Does the study add value?	Total (/10)	Grade
1. Mahon and Hevey (2021)	YES	YES	YES	PARTLY	YES	YES	YES	PARTLY	YES	YES	9/10	HIGH
2. Wiklund et al (2019)	YES	YES	YES	YES	YES	NO	YES	PARTLY	YES	YES	8.5/10	HIGH
3. Easton et al (2018)	YES	YES	YES	PARTLY	YES	YES	PARTLY	YES	YES	YES	9.5/10	HIGH
4. Burnette et al (2017)	YES	YES	YES	YES	YES	NO	PARTLY	YES	YES	YES	8.5/10	HIGH
5. Baker et al (2019)	PARTLY	YES	YES	NO	NO	NO	NO	YES	YES	YES	5.5/10	MODERATE
