# Supplementary material for: The social media diet: A scoping review to investigate the association between social media, body image and eating disorders amongst young people
Source: PLOS Glob Public Health. 2023 Mar 22;3(3):e0001091. doi: 10.1371/journal.pgph.0001091 (PMC10032524; doi:10.1371/journal.pgph.0001091)
Supplement: S1 Data — Data categorisation tables for the synthesis of results. (PDF) [file pgph.0001091.s007.pdf]

S7. Data categorisation tables for the synthesis of results

The four sections below present detailed categorisation of Exposures, Outcomes, Mediators, and Moderations. Anomalies to the trend are indicated in red.

EXPOSURES

Table A: Social Media activities

Author (s)	Study type	Findings	Comments
**Time (n=7)**			
Stein et al (2021)	CS	Time alone was not associated with body esteem and dietary restraint	Suggestive of specific uses of IG as more important
Scully et al (2020)	CS	Time alone on FB was not associated with BID – only via social comparison & internalization of ideal	Not just FB use
Wilkcsh et al (2020)	CS	Time mediated the relationship for highly visual SM usage	Large sample size
Yang et al (2020)	CS	Excessive time (+3 hours) on SM resulted in BID for adolescent girls but was mediated by comparison and thin/fit ideal	
Murray et al (2016)	CS	Excessive time on SM → diminished weight and appearance esteem → restrained eating for males and females	Did not look at mediators
Cohen et al (2017)	CS	Time alone not significantly related – only for appearance focused SM usage when mediated by thin ideal internalization and body surveillance	
Kaewpradub et al (2017)	CS	Time on SM → binging, purging, use of laxatives and crash dieting	Thailand<br>Did not look at mediators
**Frequency (n=2)**			
Vall-Roque (2021)	CS	Higher frequency of SM usage during COVID-19 lockdown was significantly associated with BID and drive for thinness amongst p's aged 14-24	Large sample size<br>Spain – not necessarily generalisable

Bennett et al (2020) *	EMA	Frequency of SM sites visited was predictive of BID, but time spent was not	EMA study design- higher ecological validity but less comparable to other study designs
**Appearance focused social media platforms (n=3)**			
Marengo et al (2018)	CS	Appearance focused SM use for >2hours/day (particularly IG and SC) was associated with BI concerns and internalizing symptoms	
Cohen et al (2017)	CS	Appearance focused IG use → BI concerns in young women	
Wilkcsh et al (2020)	CS	Time spent on image orientated platforms = DE cognitions and behaviours for adolescent girls and boys	Large sample size
**Investment in appearance related activities (n =17)**			
1. Selfie posting (n =3)			
Baminiwatta et al (2021)	CS	Posting photos of the self → BID for the whole sample	Sri Lankan study<br>Not necessarily generalisable – UN complex
Lonergan et al (2020)	CS	Avoidance of posting selfies, photo manipulation and investment in other's photos → greater likelihood of meeting criteria for clinical/subclinical ED	Large sample size (4,209)<br>Adjusted for Sociodemographic variables
Baker et al (2019)	Q	Even if you're ugly in real life, if you're cute on Instagram, I feel like it doesn't matter<br>"When I'm feeling pretty down sometimes, I'll post a selfie, or a lot of selfies. When you get likes it feels good, accomplished"	
2. Photo manipulation (n =7)			
Baminiwatta et al (2021)	CS	Photo manipulation → BID amongst girls only	Comparable rates to Australian samples
Gioia (2020)	CS	Investment in likes and manipulation of photos → self-objectification which was moderated by body shame	Low quality
Verrasto et al (2020)	CS	Editing photos → greater internalised stereotypes of beauty, BI anxiety, and fear of	

		negative evaluation	
Wick and Keel (2020)	MM- experimental & CS	Assigned to posting edited photos → increased anxiety and restrict food intake	Just 5 minutes- brief editing
Chang et al (2019)	CS	Photo posting → positive body esteem	Receiving positive feedback as a confidence boost<br>Different tools used to assess BI
Kleemans et al (2018)	MM- experimental & survey	Exposure to manipulated (desirable) photos → BID with low awareness of edits	Small sample size<br>Low quality
Limniou et al (2021)	MM- experimental & CS	IG use and posting → higher state self esteem	
3. Likes (n =7)			
Limniou et al (2021)	MM- experimental& CS	Investment in likes associated with BID, low self-esteem & low mood	Homogenous sample<br>Small<br>Low quality
Mahon and Hevey (2021)	Q	Emphasis placed on investing to look like others, seeking likes and feeling unworthy<br>**"on IG you have to look perfect because you can see how many likes you get and people feel pressured. They want more likes"**	Small sample
Lonergan et al (2019)	CS	SM photo manipulation and investment in feedback → BID	Directionality not assumed
Tiggemann et al (2018)	Experimental	Investment in likes → facial dissatisfaction but NOT BID	Small homogenous sample<br>Different tools<br>Looking at other people's photos rather than their own<br>Passive viewing
Howard et al (2017)	CS	Investment in feedback → greater BID & DE pathology for entire sample	
Baker et al (2019)	Q	Heavy investment in likes and comments, sending photos to friends, deleted photos without likes, edited<br>"**It's all about aesthetics"**<br>**"you can choose what to post, and control people's perceptions of you**	Homogenous sample

		**online”**	
Cavazos-Rehg (2020)	MM	**“I get more likes on my photos when I post a picture that accentuates a certain part of [my] face or body… I sometimes post about how unhappy I am with my appearance and people post nice things about me.**	

Table B: Social Media Trends

Author (s)	Study type	Findings	Comments
**Fitspiration (n=8)**			
Limniou et al (2021)	MM-Experimental & CS	Exposure to fitspiration = low self-esteem, low mood but no impact on body dissatisfaction	Low ecological validity – only exposed to 5 photos and no captions (guilt promoting- part of fitspiration)<br>Small sample size – underpowered<br>Low quality
Krug et al (2020)	EMA	Fitspiration exposure → pressure to attain ideal but no impact on mood or disordered eating	Low ecological validity- only viewed 1 image 6x per day
Prichard et al (2020)	Experimental	Fitspiration imagery → negative mood and BID and no difference in exercise behaviour	
Raggatt et al (2018)	MM	High risk group (17.7% risk of ED, 17.4% psychological distress, 10.3% addictive exercise)<br>Positive: motivation goal setting, community<br>Neg: anxiety, hampering ED recovery, pressure to eat clean<br>**"it has slowed my recovery from an ED, it can cause anxiety and hopelessness to know that I will never look like 'fitspiration people'**<br>**"it makes me upset that I don't feel good enough to start with"**	All followed fitspiration content – certain type of individual
Tiggemann and Andenberg (2020)	Experimental	Bare-chested images of hyper muscular males → BID	Short exposure<br>Male only<br>Not via comparison
Easton et al (2018)	Qualitative	Mixed findings: motivating, healthy recipes but comparisons, dangerous content, mental health, weight loss products, feelings of guilt, extreme meal plans, binging<br>"**you lose sight f the goal of actually trying to become healthy rather than just**	Small female sample. Does not distinguish between those who find it motivating and those who

		**looking good for pictures on social media"**<br>**"people are putting up their best photos for a reason, and it's not real life"**	find it damaging
Dignard and Jarry (2021)	Experimental	Fitspiration and thinspiration → BID relative to control group travel images<br>The wolf in little red riding hood<br>Protective effects not implemented – less awareness	Suggestive of causality<br>Equally damaging effects
Wiklund et al (2019) *	Qualitative	A fit, good-looking body = social status, healthism, #neverrest, fed with ideals, appearance as an indicator of social status, extreme exercise diets promoted by those with influence, an unfinished body	
**Thinspiration and pro-ED content (n=3)**			
Fitsimmons-Craft (2020)	CS	96% followed thinspiration→ 86% met criteria for clinical/subclinical ED and 71% and 65% reported symptoms for depression and anxiety<br>Surrounding selves with content, norm, emaciated people	High risk sample<br>Not representative<br>Self-report questionnaire – bias or desirability
Tan et al (2016)	CS	High DE scores→ sought out ED content especially on Youtube, weight loss and hiding an ED<br>Anonymity, masking an ED, tips to go hungry – food content	Clinical setting
Cavazos-Rehg (2020)	CS	Tips on being skinny, thinspo and bone thin girls<br>fixating on body<br>**"it makes me want to work out to look like the models or people in the photos or videos" "motivate myself to get skinnier"**<br>"Thinspo accounts are dangerous. They post photos of bone-thin girls, and it definitely turns my focus to my body in negative ways" "triggers me into restricting and self-hatred"	

OUTCOMES

Table C: Eating Disorder Pathology

Author (s)	Study type	Findings	Comments
**Clinical/ (n=5)**			
Lonergan et al (2020)	CS	SM investment associated with criteria for meeting clinical or subclinical EDs and night eating syndrome	
Aparicio-Martinez et al (2019)	CS	SM use significantly associated with DE – 28.5% DE attitudes, 38.7% BN symptoms and 20.7% dieting	EAT-26 questionnaire- gold standard
Puccio et al (2016)	CS longitudinal	BID → BN, depression, and restrained eating	
Fitzsimmons (2020)	CS	96% followed thinspiration content, 84% met criteria for clinical/subclinical ED	Extremely high – high risk population
Saunders and Eaton (2018)	CS	Significant association between SM use and BED and cognitive restraint	Large sample size
**Subclinical/OSFED (n=2)**			
Wiklund et al (2019)	Qualitative	Fitness fixation, extreme exercise, and obsessions with healthy food – orthorexia symptomatology – need to eat clean	Small sample<br>Not measuring ED explicitly
Turner and Lefevre (2017)	CS	IG use significantly associated with ON (49% prevalence vs <1% general population) – demonise certain foods	Biased recruitment – fitness pages<br>Legitimate scale used
**DE pathology (n=12)**<br>*Food control, dieting, laxative use, dietary restraint, compulsive exercise*			
Ding and Xu (2021)	CS	Social comparisons on SM → emotional eating amongst girls and boys	
Stein et al (2021)	CS	SNS (IG) → increased risk for DE amongst males and females	
Choukas-Bradley (2020)	CS	Comparisons on SM → DE for girls only	

Krug et al (2020)	EMA	Exposure to fitspiration → no impact on mood or DE pathology	Low ecological validity
Wilkcsh et al (2020)	CS	SM → DE behaviours including meal skipping and extreme exercise – reported by 51.7% girls and 45% boys	Self-report
Rodgers et al (2020)	CS	SM use associated with BID, dietary restraint and muscle building behaviours for girls and boys	
Wick and Keel (2020)	MM	Photo manipulation → ED symptomatology & anxiety	EAT-26 scale used
Raggatt et al (2018)	MM	Association between fitspiration on SM & ED risk (17.7% high risk), psychological distress (17.4%) & addictive exercise (10.3%)	Small sample size
Kaewpradub et al (2017)	CS	Time on SM → binging, purging, use of laxatives and crash dieting	Thailand
Howard et al (2017)	CS	Investment in peer feedback → DE for entire sample	
Santarossa (2017)	CS	SM activities (investment) → higher ED symptoms	
Murray et al (2016)	CS	Excessive time → emotional eating (females) and restrained eating (both genders)	
**ED maintenance or recovery (n=2)**			
Tan et al (2016)		EDs sought out content on YT: weight loss, tips on hiding an ED<br>50% reported that it helped to maintain their ED<br>18.2% AN, 23.6% binging, BN, 9.1%	Clinical setting
Cavazos-Rehg et al (2020) **	MM- CS & qualitative	Some engaged with SM for ED recovery & seeking treatment (2.9%), community – 97% - triggered ED<br>BUT more often was to restrict diet, and lose weight, trigger ED behaviour<br>**"They hinder recovery and reinforce the thin ideal"**<br>**"When I get really hungry, I do into these sites to get a little extra motivation to not eat just for a bit longer"**<br>Normalising ED behaviours	Self-report

Table D: Body Image concerns

Author (s)	Study type	Findings	Comments
**BID (n=36) including feelings of body shame, low self-esteem and body related anxiety**			
33 demonstrated a significant relationship between various forms of SM usage and BID – relatively homogenous finding<br>Exceptions –<br>Krug (2020) - low ecological validity<br>Chang et al (2019)- posting and positive outcomes (confidence boost)<br>Tiggemann et al (2018)- number of likes – looking at other people's photos rather than their own			
**BI as preceding DE pathology (n= 5)**			
Yao et al (2020)	CS	BI → SNS appearance comparisons → restrained eating	Hypothesised
Murray et al (2016)	CS	SNS → low body esteem → ED pathology	Hypothesised
Marengo (2018)	CS	SNS → BIDI → ED pathology & internalising symptoms	Hypothesised
Puccio et al (2016)	Observational longitudinal	FB use → BID → BN symptomatology prospectively	One-month follow up – short
Saunders and Eaton (2018)	CS	SNS→ BID → ED pathology	Hypothesised

Table E: Mental health comorbidities

Author (s)	Study type	Findings	Comments
**Mood (n= 4)**			
Krug et al (2020)	EMA	Fitspiration → no effect on mood	Low ecological validity
Prichard et al (2020)	Experimental	Fitspiration → higher negative mood relative to control travel images	
Easton et al (2018)	Qualitative	Negative effects on emotional wellbeing	Small sample size
Brown and Tiggemann (2016)	Experimental	Attractive celebrity and peer images → negative mood	
**Anxiety and Depressive symptoms (n=5)**			
Raggatt et al (2018)	CS	17.7% reported high levels of psychological distress	
Choukas-Bradley et al (2020)	CS	Association between comparisons on SM & higher depressive symptoms for girls and boys	
Fitzsimmons-Craft (2020)	CS	71% sample reported symptoms consistent with depression and 65% with anxiety (84% met criteria for clinical or subclinical ED)	Self-report<br>High<br>100% female
Wick and Keel (2020)	MM	Posting edited photos significantly associated with anxiety but not depressive symptoms	
Marengo et al (2018)	CS	Significant association between SM usage and internalizing symptoms	

MEDIATORS

Table F: Mediators

Author (s)	Study type	Findings	Comments
**Thin/fit ideal internalisation (n=12)**			
Jarman et al (2021)	CS	SM use → BID and low wellbeing via mediating pathways of thin/fit ideal for girls and boys	Large sample size
Limniou et al (2021)	MM-Experimental & CS	Fitspiration images → fit ideal internalisation reduced after viewing fitspiration images	Low ecological validity- 5 images without captions
Rodgers et al (2020)	CS	SM usage → BID via internalisation of the ideal and comparison	Large sample size
Scully et al (2020)	CS	Appearance SM usage → BID via social comparison and internalization of the ideal	
Tiggemann and Andenbeg (2020)	Experimental	Exposure to bare chested fitspiration photos → BID but not via fit/muscular ideal internalisation	Male only<br>Different mechanisms
Verrastro et al (2020)	CS	Edit photos → greater internalisation of ideal → BID	Large sample
Aparicio-Martinez (2019)	CS	Problematic SM usage → BID and DE, strong desire for ideal body	
Wiklund et al (2019)	Qualitative	Failing to meet the ideal → alienation, imperfection<br>Pressure to adhere to a predefined ideal<br>**"you are fed with their ideals, it gets to you, without being aware"**<br>Girls – remaining slim but toned and not too muscular	Small sample
Cohen et al (2017)	CS	Appearance SM use → BI concerns via thin ideal internalisation and body surveillance	
Baker et al (2019)	Qualitative	Pressure to adhere to ideal – describe many different shapes- flawless makeup, stick thin, hourglass figure<br>"**we both followed the girl I mentioned that's just so pretty. We will**	Small sample

		**screenshot her pictures and try to mimic them"**	
Yang et al (2020)	CS	Excessive SM use → BID via internalisation of thin/fit ideal and upward appearance comparisons	
Mahon and Hevey (2021)	Qualitative	Picking flaws and self-blame for not adhering to the ideal for males and females	Small sample
**Appearance comparisons (n=21)**			
Dignard and Jarry (2021)	Experimental	Viewing fitspiration & thinspiration → BID via appearance comparisons	
Ding and Xu (2021)	CS	SM → emotional eating mediated by upward comparisons and anxiety for both genders	
Jarman et al (2021)	CS	Appearance SM usage → BID and low wellbeing via appearance comparisons and ideal internalisation	
Jiotsa (2021)	CS	Association between frequency of comparisons and BID & drive for thinness	Large sample size<br>Majority female
Mahon and Hevey (2021)	Qualitative	Despite awareness, engaged in comparisons with others – SM influencers, celebrities<br>"**Why can't I look like that, why can't I be that person?'**<br>**'We always compare ourselves to the people we see on SM, so we don't see their flaws'**	Small sample
Rodgers et al (2020)	CS	SM ideal internalisation and higher upward appearance comparison → BID	
Rosseau et al (2017)	Observational longitudinal	Comparisons on FB not predictive of increases in BID 6-months later	Other mechanisms at play<br>FB outdated amongst younger samples
Scully et al (2020)	CS	Significant relationship between appearance-based SM and BID via social comparison and internalisation of the ideal	
Tiggemann and Andenberg (2020)	Experimental	Social comparison did not mediate the relationship between fitspiration images and BID	Male sample<br>Singapore
Yang et al (2020)	CS	Excessive SM use → BID via upward appearance comparisons	

Yao et al (2020)	CS	SM usage → comparisons → shame → restrained eating	
Chang et al (2019)	CS	Photo browsing on IG → negative BE via appearance comparisons	
Saunders and Eaton (2018)	CS	Positive correlation between upward comparison and DE outcomes<br>Stronger for IG and SC	Hispanic sample
Brown and Tiggemann (2016)	Experimental	Exposure to celebrity & peer images → BID via upward comparison<br>Didn't matter if it were celebrities or peers	Only partly agrees with SCT
Puccio et al (2016)	Observational longitudinal	Social comparisons mediated relationship between FB use & BN symptomatology	
Tan et al (2016)	CS	70.9% of sample reported comparing themselves to others on SM	Clinical sample – high risk
Baker et al (2019)	Qualitative	Comparison to others to degree that p's would imitate photos and recreate	University students
Burnette et al (2017)	Qualitative	Despite SM literacy, some girls still compared with others<br>Could appreciate beauty without jealousy	6 focus groups
Cavazos-Rehg (2020)	MM	**"I obsessively compare my body and lifestyle to others"**	
Easton et al (2018)	Qualitative	**"When I see fitness accounts where all the girls are like svelte and toned, I'm like oh, it's hard to love me when I look like this"**	
Baker et al (2019)	Qualitative	**"I thought it was only celebrities that looked like that, but I think it's everybody, so I'm the ugly one basically"**	
**Self-objectification (n=6)**			
Saunders and Eaton (2018)	CS	Body surveillance mediated the relationship between SM use, BID and DE outcomes	
Cohen et al (2017)	CS	Body surveillance mediated the relationship between appearance SM use and BI concerns for women	
Baker et al (2019)	Qualitative	Internalising 3rd person perspective of the self, worrying about how one looks to others	
Mahon and Hevey (2021)	Qualitative	Picking out flaws in photos, hating photos, self-criticism<br>**The more you look at the photo you're like 'God I hate it' you see things that other people wouldn't see and you're like 'I hate everything about it'."**	
Choukas-Bradley et al (2020)	CS	Higher scores on ARSMC → DE amongst 96% girls and 91% boys	Scale developed to assess degree

		"Imaginary audience"	to which people care about online appearance
Cavazos-Rehg (2020)	MM	**"I get more likes on my photos when I post a picture that accentuates a certain part of [my] face or body"**	
**Other (n= 2)**			
		Anxiety (n=1)	
Ding and Xu (2021)	CS	Social comparisons → appearance anxiety → emotional eating	China<br>Plausible that appearance anxiety precedes both social comparisons and emotional eating
		Self-schema and self-discrepancy (n=1)	
Ahadzadeh et al (2017)	CS	IG photo exposure associated with BID but mediated by self-schema → self-discrepancy	Novel finding

MODERATORS

Table G: Biological

Author (s)	Study type	Findings	Comments
**Gender (n=18)**			
Difference (n= 14)			
Ding and Xu (2021)	CS	Females more sensitive to appearance comparisons and showed more emotional eating tendencies	Equal ratio of girls to boys<br>Micro-blogs – different SM form
Limniou et al (2021)	MM- Experimental & CS	Exposure to fitspiration images- males internalised fit ideal more but females showed lower mood post exposure	
Mahon and Hevey (2021) *	Qualitative	Girls placed greater emphasis on comparisons, awareness of editing and had lower SE vs boys who saw muscular ideal as motivating<br>**B – "I'm grand just the way I am"**	Stigma for boys<br>Majority female sample
Stein et al (2021)	CS	Females held unfavourable views of own bodies and others but IG → DE equally for males and females	Online sample
Choukas-Bradley (2020)	CS	Higher appearance comparisons → DE in boys only	Alternative measure
Gioia (2020) *	CS	Young women displayed higher levels of body shame, invested more in self photos	
Rodgers et al (2020)	CS	Gender moderated the relationship between SM & muscular ideal internalisation for boys only *	
Tiggemann and Andenberg (2020)	Experimental	Fitspiration → BID relative to control but not via social comparison and muscular ideal internalisation	Novel<br>Other mechanisms for men?
Verrastro et al (2020)	CS	Females showed higher levels of BI anxiety and internalised the ideal of beauty more but both genders feared negative evaluation from peers	
Easton et al (2018)	Qualitative	Damaging fitspiration effects emerged as stronger for girls<br>**I reckon it probably negatively affects boys, but they don't express it. If a boy did he'd probably be called a wimp"**	

Kaewpradub et al (2017)	CS	Female p's demonstrated greater BID and DE pathology	Thailand
Murray et al (2016)	CS	SM → emotional eating for females only	More invested emotionally<br>Self-report
Wilksch et al (2020)	CS	Girls posted more photos of food and displayed higher DE cognitions	Low quality
Cavazos-Rehg (2020)	CS	Subclinical ED group contained high proportion of transgender participants – risk - warrants further research	
No difference (n=4)			
Baminiwatta et al (2021)	CS	Selfies and BID for entire sample	Low quality
Jarman et al (2021)	CS	Gender not a significant moderator for appearance SM use & BID but boys more likely to internalise the muscular ideal	
Santarossa and Woodruff (2017)	CS	No difference between genders for SM investment, BI concerns and ED pathology	
De Vries et al (2019)	CS	SM → BID for male and female adolescents	Different scales, younger age group= more susceptible?
**BMI (n=5)**			
Baminiwatta et al (2021)	CS	BMI directly associated with BID	Sri Lanka<br>Low quality
Rodgers et al (2020)	CS	BMI directly associated with BID but also heightened comparison and SM ideal internalisation	
Yao et al (2020)	CS	High BMI → more BI comparisons but low BMI → more shame and restrained eating	Inconsistent- lower BMI so closer to the ideal- behaviours as more in reach<br>Very low average BMI
Jiotsa (2021)	CS	BMI was not a moderator between SM use and ED pathology	193 ED patients- skewed findings – lower BMI
Easton (2018)	Qualitative	**"If you're a bigger size it can make you feel horrendous, it can make you feel**	

		**completely alien, and that you shouldn't look like that"**	
**Age (n=1)**<br>*Inconclusive*			
Vall-Roque (2021)	CS	SNS frequency (appearance focused) → stronger for younger p's 14-24	Large sample
**Race/Ethnicity (n=3)**<br>*Inconclusive*			
Howard et al (2017)	CS	Race was not a significant moderator, but Black women reported marginally less DE and higher state body satisfaction	Large sample size
Wick and Keel (2020)	MM	Editing & posting photos – stronger effect for White & Asian participants	77% White
Dignard and Jarry (2021)	Experimental	Non-white participants – fitspiration & BID was not mediated by appearance comparisons	SM images not reflective of themselves<br>Canadian sample
**Hormones (n=1)**<br>*Inconclusive – warrants further research*			
Aparicio-Martinez (2019)	CS	High levels of prenatal testosterone → decreased association between SM use, BID and DE behaviours	

Table H: Cognitive

Author (s)	Study type	Findings	Comments
**Pre-existing BI concerns (n=6)**			
		BID/body shame/low self-esteem	
Gioia (2020)	CS	Body shame predicted image control and problematic SM use in males and females	CS so cannot assume directionality<br>Low quality
Rousseau (2017)	Observational longitudinal	State BID predicted comparisons on FB 6-months later	Indicating that dissatisfied individuals are more susceptible
Ahadzadeh et al (2017)	CS	Low self-esteem → strengthened relationship between SM, BI & ED pathology	
Kim and Park (2016)	Experimental	Low self-esteem → strengthened relationship between SM comparisons and BID	
		Body appreciation	
Yao et al (2020)	CS	Body appreciation buffered the relationship between SM usage, BS and restrained eating	
Lonergan et al (2019)	CS	Self-compassion did not moderate the relationship between SM photo manipulation and BID	Discrepancy in tools used
**Underlying risk of ED (n=5)**			
Jiotsa (2021)	CS	High SCOFF scores = higher BID, drive for thinness and comparisons	Large sample size<br>1331
Cavazos-Rehg (2020)	MM- CS and open questions	Clinical/ED subgroup- stronger relationship between wanting to be thin and restricting food intake	
Fitsimmons-Craft (2020)	CS	96% followed thinspiration content and 86% met criteria for an ED – strong relationship between ED content and diagnoses	CS- cannot assume directionality
Verrastro (2020)	CS	Adolescents with greater internalised standards of beauty report higher BI anxiety, pressure to adhere to ideal & fear of negative evaluation from others	CS

Tan et al (2016)	CS	Those with higher ED scores sought out certain damaging ED content: weight loss info, tips on hiding an ED, finding support	
**Other: personality traits (n=2)**			
Puccio (2016)	CS	Stronger relationship between SM & BN for those with personality trait sociotrophy	
Kim and Park (2016)	Experimental	Exposure to attractive peers → BID with larger effect size of those with high appearance comparison tendencies	

Table I: Socioenvironmental

Author (s)	Study type	Findings	Comments
**SM literacy (n=4)**			
Burnette (2017) **	Qualitative	SM education at school buffered effect. Still compared but did not lead to significant BI concerns or ED pathology<br>Learnt about artificiality, self-acceptance & body diversity<br>**"I don't need people to tell me I'm pretty on social media"**	Anomaly<br>Small sample size
Mahon and Hevey (2021)	Qualitative	Contrasts with Burnette^ less SM literacy and greater impact	Anomaly to general trend
Easton et al (2018) **	Qualitative	Participants were critical of SM yet still struggled to switch off from posts & avoid negative feelings about the body and eating concerns	Older sample – maybe had not had the same nurturing school environment
Kleemans (2018)	MM- Experimental & survey	Exposure to edited photos → BID<br>Low awareness of editing practices, rated as more attractive	Low SM generally
**Parental relationships (n=1)**			
DeVries et al (2019)	CS	SM → BID but weaker for adolescents with a positive mother-adolescent relationship	Needs further research
**Life events (n=2)**			
Easton (2018)	Qualitative	Fitspiration affected emotional state more when mood was low combined with sensitivity following stressful life events	Insight
Cavazos-Rehg (2020)	MM	"**They don't always have a substantial effect on me but at times of weakness they can push me into relapse"**	
