# Supplementary material for: The social media diet: A scoping review to investigate the association between social media, body image and eating disorders amongst young people
Source: PLOS Glob Public Health. 2023 Mar 22;3(3):e0001091. doi: 10.1371/journal.pgph.0001091 (PMC10032524; doi:10.1371/journal.pgph.0001091)
Supplement: Attachment — Submitted filename: Response to Reviewers_PGHP_D_22_01315_13Jan2023.docx [file pgph.0001091.s008.docx]

Response to Reviewers: Manuscript ID PGPH-D-22-01315

[Authors’ responses in blue.]

Journal Requirements:

1. Please review your reference list to ensure that it is complete and correct. If you have cited papers that have been retracted, please include the rationale for doing so in the manuscript text, or remove these references and replace them with relevant current references. Any changes to the reference list should be mentioned in the rebuttal letter that accompanies your revised manuscript. If you need to cite a retracted article, indicate the article’s retracted status in the References list and also include a citation and full reference for the retraction notice.

We have reviewed the reference list and have not made any changes to it as we believe it is complete and correct.

2. Please provide separate figure files in .tif or .eps format only and remove any figures embedded in your manuscript file. Please also ensure that all files are under our size limit of 10MB.

We have converted figures into .tif format and re-submitted these as separate files.

Reviewers' comments:

Reviewer #1: Overall, this is a well-written manuscript with rigorously methodology.
We thank the reviewers for their positive feedback and constructive comments.

Specific comments are follows:

#Figure 1: PRISMA flow diagram
Please explicitly mention the "other sources" from where you have identified three additional records (wherever applicable).
We have amended figure one to clarify that “other sources” refers to background reading material.
#Recommendations and future research
page 29, line 597: Societal and health system level: The recommendations for health system level seems to be vague and focused mainly on clinical, policy, and global level. Please provide the recommendation from lowest tier to highest tier of health care system. It is not necessary to make different sections but the recommendations should be clear and detailed.

Thank you for this useful comment. However, it will be difficult for us to add in specific recommendations for each tier of the health care system. This is because our review was exploratory, and this literature is in its infancy – we therefore do not have a sufficiently detailed evidence base from which we can outline specific recommendations for health systems. We appreciate that this section therefore remains vague, but we took care to point out the exploratory nature of recommendations at the start of the section. We anticipate that future review work focussed on healthcare systems can use our paper as a starting point for providing more detailed recommendations. We hope that this does not diminish the overall quality of our research article.

#Others
page 5, line 87, Table 2: check spelling of psychosocial

Corrected. Thank you for spotting it!

Reviewer #2: The subject is fascinating and significant from the standpoint of public health. But improvements are required to make it better. The suggestions are as follows:

• Please include the terminologies/keywords used for the search

These are already in a table (Table 3 previously, now Table 4 to reflect conversion of Panel 1 to Table 4) just below the description of the search strategy.

• Line 47: There are more than six categories in table 1. What are the other categories? This is not clear. Please clarify

We apologise for the lack of clarity. Eating disorders are split into two main categories – CLINICAL and SUB-CLINICAL / OFSED. Within CLINICAL there are six types and SUBCLINICAL covers others. The table already makes this distinction by using merged columns, but we have now made this clearer putting the merged column headings in capital letters and formatting line spacing. We hope this is now much clearer.

• Table 2: The information presented here looks more suitable for a figure than a table.

Thank you for this suggestion. We considered presenting this as a figure, but due to the large amount of text in it we decided to keep it as a table.

• Line 134: Why is named as a Panel. Can’t this be a table?

We used the label Panel as it presents background information relevant to our methodology rather than specific methods or tasks we have undertaken. However, we take the suggestion and have re-named it as Table 3 and changed the ordering / numbering of tables across the manuscript to reflect this.

• It will be easy for the reader to understand if the summary of the theoretical framework can be depicted in the form a diagram within the article.

Thank you for this suggestion. While the theoretical framework would be very useful as a diagram in the Methods section, it would be somewhat repetitive because Figure 2 in the Results already uses much of the theoretical framework to present our results and synthesis. Therefore we will retain the summary in the Methods section and have decided not to convert it into a diagram. However, one of the main sources that we drew on does indeed lay out many of these theories in a diagram, in the paper by Rodgers (reference 43). We hope that this is acceptable and does not diminish the overall quality of our article.

• It may be better if the PRISMA checklist was included in the text itself rather than in the supplementary files.

The PRISMA checklist will indeed be in the main body of the article, and is already captioned as Figure 1 in the original submission below the Study Selection paragraph. However, since the journal requires figures to be submitted separately, we did not embed the PRISMA flowchart into the word document.

• The results part quite lengthy and has a lot of sections and sub-sections. I will be easier to follow if a few lines are written at the beginning of the results describing how you have divided the findings.

Thank you for this excellent suggestion. We have implemented it.
